# Quantifying citrate-enhanced phosphate root uptake using microdialysis

**DOI:** 10.1007/s11104-019-04376-4

**Published:** 2019-12-05

**Authors:** D. M. McKay Fletcher, R. Shaw, A. R. Sánchez-Rodríguez, K. R. Daly, A. van Veelen, D. L. Jones, T. Roose

**Affiliations:** 1 Bioengineering Sciences Research Group, Department of Mechanical Engineering, School of Engineering Sciences, Faculty of Engineering and Physical Sciences, University of Southampton, University Road, Southampton, SO17 1BJ UK; 2 Environment Centre Wales, Bangor University, Deiniol Road, Bangor, Gwynedd LL57 2UW UK; 3 Agronomy Department, University of Córdoba, Campus de Rabanales. Edificio C4 Celestino Mutis, 14071 Córdoba, Spain; 4 SoilsWest, UWA School of Agriculture and Environment, The University of Western Australia, Perth, WA 6009 Australia

**Keywords:** Method, Modelling, Nutrient uptake, Phosphorus mobilisation, Soil solution

## Abstract

**Aims:**

Organic acid exudation by plant roots is thought to promote phosphate (P) solubilisation and bioavailability in soils with poorly available nutrients. Here we describe a new combined experimental (microdialysis) and modelling approach to quantify citrate-enhanced P desorption and its importance for root P uptake.

**Methods:**

To mimic the rhizosphere, microdialysis probes were placed in soil and perfused with citrate solutions (0.1, 1.0 and 10 mM) and the amount of P recovered from soil used to quantify rhizosphere P availability. Parameters in a mathematical model describing probe P uptake, citrate exudation, P movement and citrate-enhanced desorption were fit to the experimental data. These parameters were used in a model of a root which exuded citrate and absorbed P. The importance of soil citrate-P mobilisation for root P uptake was then quantified using this model.

**Results:**

A plant needs to exude citrate at a rate of 0.73 μmol cm^−1^ of root h^−1^ to see a significant increase in P absorption. Microdialysis probes with citrate in the perfusate were shown to absorb similar quantities of P to an exuding root.

**Conclusion:**

A single root exuding citrate at a typical rate (4.3 × 10^−5^ μmol m^−1^ of root h^−1^) did not contribute significantly to P uptake. Microdialysis probes show promise for measuring rhizosphere processes when calibration experiments and mathematical modelling are used to decouple microdialysis and rhizosphere mechanisms.

**Electronic supplementary material:**

The online version of this article (10.1007/s11104-019-04376-4) contains supplementary material, which is available to authorized users.

## Introduction

Low phosphate (P) availability in soil is often one of the most severe constraints to crop production worldwide (Barber 1995; Vitousek et al. 2010). This is particularly pertinent in low income countries where farmers have insufficient capital to replenish their soil P supply with synthetic P fertilisers (Sanchez 2002). Under-fertilisation is not the only reason for low P availability in soils. P strongly adsorbs to soil particle surfaces (e.g. Fe/Al oxides) and can become immobilised by microbes, both of which decreases the amount of P directly available to plants (Barber 1995; Oburger et al. 2011a).

To overcome P limitation in soils, plants have evolved a range of strategies to manipulate the soil environment to increase P bioavailability. Of these, the exudation of organic acids (e.g. citrate and oxalate) by roots of many species has been shown to promote P mobilisation (Chen and Liao 2016). This has been shown to occur via four main mechanisms: 1) the co-excretion of H^+^ and organic acid anions to lower the pH of the soil solution and promote ligand competition and desorption of inorganic P held on Fe and Al mineral surfaces, referred to as ‘specific adsorption’; 2) release of organic acids leading to the complexation of cations in solution (e.g. Ca^2+^) which then promotes P mineral dissolution; 3) the direct attack and removal of cations on mineral surfaces (e.g. apatite) by organic acid anions leading to inorganic P release, referred to as ‘ligand-promoted dissolution’; and 4) solubilisation of organic P (Oburger et al. 2011a). For soils in which the sorption sites have low P saturation, it was found that ligand-promoted dissolution was the dominant mechanism, while specific adsorption is dominant when sorption sites in soil are saturated with P (Oburger et al. 2011a). Typically, it is difficult to decouple these four effects from each other as they often occur simultaneously. However, specific adsorption and ligand-promoted dissolution can be decoupled from pH in experiments by counteracting pH changes caused by organic acids with the addition of strong acids or bases to achieve constant pH levels (Gerke et al. 2000a; Oburger et al. 2011a).

A number of models have been proposed to describe P solubilisation by organic acids. The simplest approach is to introduce a singular ‘solubilisation parameter’ per solubilised species (Nye 1983) to increase solubility of one species based on the concentration of the competing species. Instances of models using this approach assume all solute-soil reactions are fast and hence the bound concentration is well approximated by solution concentration (Gerke et al. 2000a; Zygalakis and Roose 2012). Competitive Langmuir reaction equations assume there is a given number of binding sites per mass of soil, which two species compete for, each occupying a given number of binding sites per ion bound. This approach introduces three additional parameters along with the rate constants. Similar to the previous approach, this model is often considered in equilibrium (Schnepf et al. 2012). Both approaches fail to capture the complex changes in soil reactions over long time periods (Barrow 1989).

Experiments used for studying the adsorption and desorption of molecules such as organic acids and P in soil can be classified into ‘equilibrium’ and dynamic experiments. For the vast majority of equilibrium experiments, a known amount of P or organic acid is added to soil, the sample is left to reach apparent equilibrium and then the amount of P and/or organic acid remaining in solution is measured (Barrow 1978; Geelhoed et al. 1998; Jones and Brassington 1998; Oburger et al. 2011b). However, these methods only assess the equilibrium behaviour of molecules in bulk soil, failing to capture the dynamic rhizosphere processes which are thought to be highly influential in plant P capture from soil. Dynamic experiments are less common when estimating P and organic acid adsorption as soil reactions are often assumed to reach equilibrium state quickly (Gerke et al. 2000a; Roose and Fowler 2004; Zygalakis and Roose 2012). Furthermore, it is difficult to analyse the dynamic processes without disrupting the in situ soil sample during measurement. Pseudo-dynamic experiments to measure P and organic acid adsorption/desorption reaction rates have been undertaken whereby different replicates are destructively sampled after a range of equilibration times (Keyes et al. 2013; Oburger et al. 2011b). These measurements may not be representative of rhizosphere processes due to variation across replicates. Consequently, estimating the significance of organic acids for alleviating P deficiency remains extremely difficult.

Microdialysis probes offer a non-destructive method for assessing soil solution ion concentrations with high spatial and temporal resolution. Microdialysis has been used to estimate nitrogen (N) availability and diffusion in soils (Inselsbacher and Näsholm 2012; Inselsbacher et al. 2011; Shaw et al. 2014); to sample Cu and Ni in soil solution (Mosetlha et al. 2007); to sample organic acids exuded by roots in soil with high temporal and spatial resolution (Sulyok et al. 2005); and P availability in soil (Demand et al. 2017). It has been argued that microdialysis probes may offer a superior method of assessing soil nutrient availability for plant-uptake due to its diffusive-based method of sampling (Inselsbacher and Nasholm 2012, Shaw et al. 2014). A further advantage of the microdialysis method is that it allows the simultaneous efflux of compounds from the dialysate and influx of solutes from the soil solution, creating a pseudo-rhizosphere (Demand et al. 2017). Demand et al. (2017) found perfusing the probes with citrate (1 mM) increased the uptake of P by the probe in an Endostagnic Luvisol soil (high water-extractable P), while in a Dystric Cambisol (low water-extractable P) little difference was seen. They also showed that the pH of the external solution did not alter probe P uptake between the ranges of 3.5 and 6.5. However, factors such as ionic strength were shown to have a large effect on the uptake rate of P by microdialysis probes (Demand et al. 2017). Furthermore, a diffusive based method may not give reliable results for nutrients which are transported primarily via advection. Thus, directly inferring plant-uptake or plant induced nutrient mobilisation using microdialysis probes may be a naïve approach; these measurements depend on a multitude of factors which can affect either the osmosis-driven mass flow movement of water in and out of the microdialysis probe or the diffusion of molecules into and out of the perfusate (Demand et al. 2017; Menacherry et al. 1992; Shaw et al. 2014). Therefore, the technique lends itself to a combined experimental-modelling approach to decouple the effects of varying microdialysis osmosis rates and in-situ rhizosphere processes from the microdialysis measurements. This approach could provide a unique method for investigating how organic acids exuded by roots affect the availability of P and potentially increase plant uptake.

In this study, we aimed to quantify the effectiveness of citrate exuded by roots on improving P mobilisation in a Eutric Cambisol soil using microdialysis probes in combination with modelling. After careful calibration of microdialysis probe influx and efflux rates, the microdialysis probes were perfused with citrate at a range of concentrations to simulate root efflux. The recovery of added isotopically labelled phosphate from the soil was used to quantify the effect of the citrate efflux on P influx. As discussed above, understanding the mechanisms of soil P mobilisation directly through experimentation is difficult and interpreting microdialysis-derived results is complex. Therefore, the experimental data was used to parameterise a kinetic model that describes the efflux of citrate from the microdialysis probe and the subsequent uptake of P. The model was then used to explore how citrate efflux affected P availability in the rhizosphere. The experimental and modelling results are discussed within both a mechanistic and an ecological context. The results offer a better understanding of small-scale in-situ rhizosphere processes.

## Materials and methods

### Soil characterisation and sampling

The soil used in this study was sampled from the Ahp horizon of a *Lolium perenne* L. dominated agricultural grassland located at the Henfaes Research Station, Abergwyngregyn, Wales, UK (53°14’N, 4°01’W). The soil is classified as a Eutric Cambisol and has a sandy clay loam texture and a fine crumb structure (Wrb 2015). Four independent soil samples (*n* = 4) were taken to a depth of 15 cm from 30 × 30 cm areas within the field. The soil was sieved to pass 2 mm and refrigerated at 4 °C until required. The properties of the soil are summarised in Table 1.

**Table 1:** General properties of the Eutric Cambisol soil used in the experiments. Measurements of crystalline and amorphous Fe and Al, and total Fe, Al and Ca taken from Oburger et al. (2011a) analysis on the same soil. CDB indicates citrate-dithionate-bicarbonate extractable (Jackson et al. 1986), AAO indicates acid-ammonium-oxalate extractable (Loeppert and Inskeep 1996). Available phosphate was extracted with 0.5 M acetic acid using a soil-to-solution ratio (SSR) of 1:10 (*w*/*v*) (Oburger et al. 2009). Values represent means ± Standard Error of the Mean (SEM). Nutrient data expressed on a dry soil weight basis

Property	Mean ± SEM
pH_(H2O)_	6.12 ± 0.05
Electrical conductivity (μS cm^−1^)	26.5 ± 0.1
Water holding capacity (g kg^−1^)	356 ± 6
Total C (g kg^−1^)	25.35 ± 1.47
Total N (g kg^−1^)	2.95 ± 0.06
Clay (%)	20
Silt (%)	37
Sand (%)	43
Crystalline Fe/Al (CBD)	
Fe (g kg^−1^)	1.4 ± 0.1
Al (g kg^−1^)	1.4 ± 0.1
Amorphous Fe/Al (AAO)	
Fe (g kg^−1^)	5.0 ± 0.1
Al (g kg^−1^)	1.6 ± 0.0
Total (Aqua regia)	
Fe (g kg^−1^)	46 ± 0.5
Al (g kg^−1^)	28 ± 0.6
Ca (g kg^−1^)	1.9 ± 0.1
Exchangeable Ca (mg kg^−1^)	501 ± 122
Exchangeable K (mg kg^−1^)	46.1 ± 12.6
Exchangeable Na (mg kg^−1^)	25.4 ± 5.1
Available P (mg kg^−1^)	22.6 ± 6.2
P sorption capacity (mg kg^−1^)	150

### Microdialysis setup

To characterise citrate P mobilisation using microdialysis, calibration of the microdialysis probes with P and citrate was required. A description of microdialysis theory and nomenclature can be found in the Supplementary Material. Two calibration experiments were designed to estimate the microdialysis probes efflux of citrate from the perfusate into the soil and the microdialysis probes influx of P into the probe at varying concentrations of citrate in the perfusate.

For all experiments described here, a WM-205u peristaltic pump (Watson-Marlow Ltd., Falmouth, UK) was used to pump citrate solutions through CMA 20 microdialysis probes (CMA Microdialysis AB, Kista, Sweeden). The probes had a 20 kDa molecular weight cut-off and polyethersulfone membrane (4 mm long, 500 μm external diameter). The pump flow rate was set to 3.3 μl min^−1^ to maximise the relative recovery of solutes from the soil (Inselsbacher et al. 2011). Dialysates were continuously collected in 1.5 ml microfuge tubes or 5 ml polypropylene vials, which were covered with Parafilm-M (Bemis Inc., Neenah, WI) to prevent evaporative losses. Prior to use, the microdialysis probes were placed in high purity water (18 MΩ resistance) and flushed with perfusate to remove any contaminants. Prior to dialysate collection, the microdialysis probes were run for approximately 10 min, to ensure the dead volume in the microdialysis probe had been flushed. Throughout the microdialysis experiments the soil moisture was maintained at 80% field capacity.

### Quantifying citrate efflux rate into the soil using microdialysis probes

Sieved field-moist soil (1.3 g; 1 g DW equivalent) was placed in a 1.5 ml microfuge tube and packed to a density of 1 g cm^−3^. A microdialysis probe was inserted into the soil using the needle and introducer supplied by the manufacturer so that the top of the probe membrane was located 5 mm below the soil surface. Sterile solutions of ^14^C-labelled citrate at concentrations 0.1, 1 and 10 mM; 1.7 kBq ml^−1^ (with pH 5.6, 4.6 and 3.6 respectively) were pumped through the microdialysis probes and the dialysate collected after 1, 2, 3, 4, 5, 6, 8 and 12 h in 5 ml polypropylene vials (*n* > 3). The amount of ^14^C-citrate remaining in the dialysate (i.e. not passed into the soil) was determined using a Wallac 1404 liquid scintillation counter (PerkinElmer Inc., Waltham, MA) following the addition of 4 ml of HiSafe-3 scintillant (PerkinElmer Inc.). The amount of ^14^C-citrate lost to the soil was calculated as the difference between the amount of ^14^C in the influent versus that in the effluent (dialysate). The volume of dialysate recovered was also recorded to confirm that there was no significant mass flow of water either into or out of the probes.

### P recovery from standard solutions using microdialysis probes

To calibrate P recovery by the microdialysis probes and to evaluate the osmotic effects of the citrate perfusate, a simple experiment was performed. Briefly, microdialysis probes were placed in 1.5 ml microfuge tubes containing 1 ml of a ^33^P-labelled KH_2_PO_4_ standard solution (0, 0.1, 1 or 10 mM, *n* = 4; 1.7 kBq ml^−1^). The microdialysis probes were perfused with solutions of citrate at concentrations of 0, 0.1, 1 and 10 mM and the dialysate collected in 5 ml polypropylene vials over a 1 h period, the amount of ^33^P in the dialysate and the volume of dialysate was determined as described above.

### P recovery from soil using microdialysis probes

Soil (1.3 g) was placed in individual microfuge tubes (*n* = 4). Subsequently, 100 μl of a ^33^P-labelled solution (1 mM KH_2_PO_4_; 370 kBq ml^−1^) was injected into the soil. The soil was mixed and left to stand at 20 °C for 24 h to approach equilibrium. A microdialysis probe was inserted into the soil as described above. Solutions of citrate (0, 0.1, 1 or 10 mM) were perfused through the microdialysis probe and the dialysate collected hourly over a 12 h period. The amount of ^33^P in the dialysate was determined using liquid scintillation counting as described earlier.

### Modelling and data fitting

#### Mathematical Model

The measurements obtained by the experiments are the result of pore scale reactions between citrate, P, soil and the microdialysis probe. Understanding these mechanisms directly through experiments is difficult. Thus, a model was constructed to aid the interpretation of the results. Linear approximations of the mechanisms were used to circumvent the danger of over-fitting the data by introducing excess parameters. In particular, the experiments do not measure P or citrate isotherms, thus we cannot accurately parameterise models including the full set of parameters. The adsorption/desorption part of the model is similar to (Zygalakis and Roose 2012) with the soil reactions maintained in their dynamic form. The model used was a kinetic model that describes the efflux of citrate into the soil and the absorption of P by the microdialysis probe. The model considers the diffusion and buffering of citrate and P, and specific adsorption between P and citrate on soil sorption sites. It is hypothesised that citrate mobilises P held on the soil, but also influences P influx rate at the probe-soil interface (Demand et al. 2017). The effect of acidity is not directly considered in the model because the effect of pH cannot be uncoupled from specific adsorption in the current experiments; instead, any additional P absorbed by the probe due acidity is attributed to the specific adsorption mechanism during the subsequent data fitting. This is a simplification of the complex relationship between pH and citrate mobilisation of phosphate, for example, at high pHs citrate can complex with calcium in the soil, reducing the effectiveness of specific adsorption (Barrow et al. 2018).

**Fig. 1:**
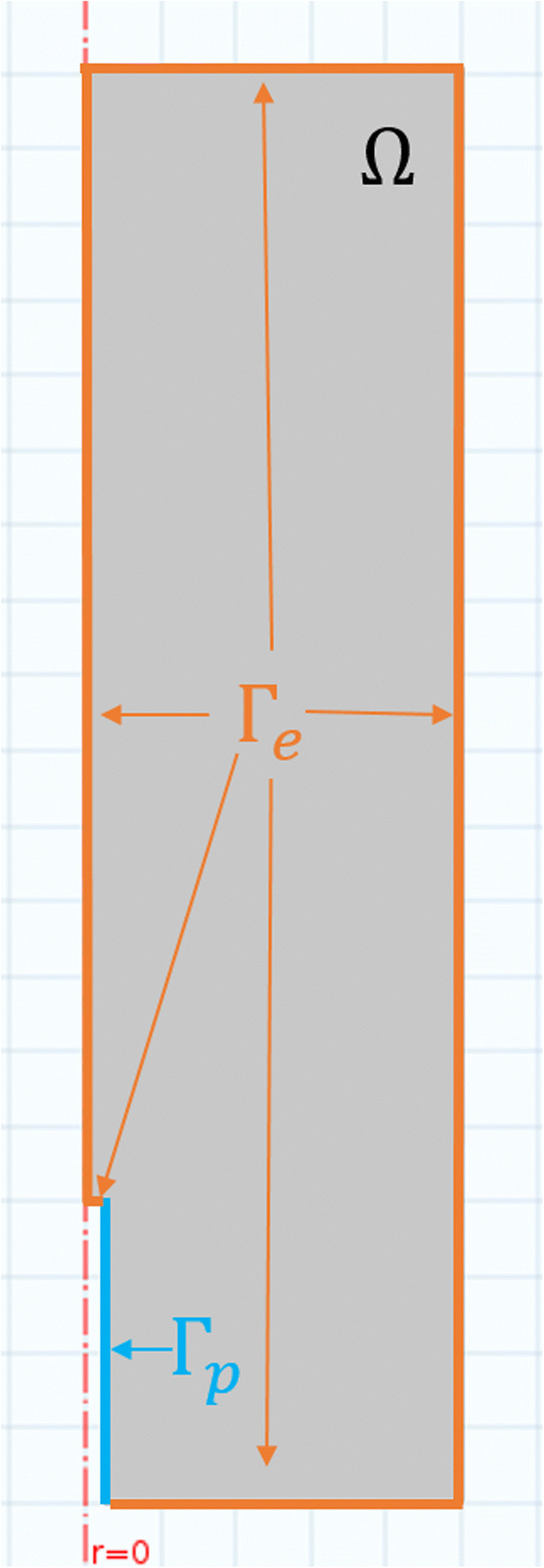
Axisymmetric representation of the domain for the model. The red-dashed line shows the axis of symmetry, Ω represents the homogenous soil within the 1.5 ml microfuge tube, Γ_p_ represents the microdialysis probe membrane, shown in blue and Γ_e_ represents the microfuge tube boundary and tip of the probe, shown in orange

The domain considered is a cylindrical region of soil with a diameter of 10 mm and height of 19.1 mm, centred about the cylindrical microdialysis probe with a diameter of 0.5 mm and height of 4 mm. These dimensions are chosen to match the experimental set up. The soil domain is denoted Ω ⊂ *ℝ*^3^, the microdialysis probe membrane is denoted Γ_*p*_ and the remainder of the boundary is denoted Γ_*e*_. The soil is assumed to be homogenous with approximate volumetric water content *ϕ*_*l*_ [m^3^ of soil solution m^−3^ of total soil] and volumetric soil solid content *ϕ*_*s*_ [m^3^ of soil solid m^−3^ of total soil]. Due to rotational symmetry of the problem, the domain can be simplified to a 2D axisymmetric representation as shown in Fig. 1; for generality, the governing equations are stated in 3D Cartesian co-ordinates, but implemented numerically as 2D cylindrical co-ordinates.

P and citrate are assumed to exist in either bound or unbound (in solution) states. In particular, *P*_*l*_(*t*, ***x***) [μmol m^−3^ of soil solution] is the P concentration in solution, *C*_*l*_(*t*, ***x***) [μmol m^−3^ of soil solution] represents citrate concentration in solution, *P*_*s*_(*t*, ***x***) [μmol m^−3^ of soil solid] is the concentration of P bound to soil particles, and *C*_*s*_(*t*, ***x***)) [μmol m^−3^ of soil solid] is the amount of citrate bound to soil particles.

P and citrate can adsorb to, and desorb from, soil particles. This was modelled using a reversible first-order chemical reaction. The process of specific adsorption promotes P desorption as more citrate is adsorbed to the soil. This was implemented by a adding a cross term to the P desorption-adsorption reaction (Eqs. (1–2)). Citrate is known to be consumed by microbes in the rhizosphere. However, sorption of citrate to soil particles causes a significant reduction in biodegradation rate (up to 99%) (Jones and Edwards 1998; Van Hees et al. 2003). Therefore, citrate in the model is allowed to biodegrade only in the soil solution. Hence, assuming conservation of mass and neglecting convective transport, the diffusion-sorption-reactions for P and citrate can be written as

1 $$ \frac{\phi_l\partial {P}_l}{\partial t}=\mathbf{\nabla}\cdotp {\phi}_l{D}_P\mathbf{\nabla}{P}_l-{\phi}_l{\beta}_1{P}_l+{\phi}_s{\beta}_2{P}_s+{\phi}_s{\beta}_3{C}_s{P}_s, $$

2 $$ \frac{\phi_s\partial {P}_s}{\partial t}={\phi}_l{\beta}_1{P}_l-{\phi}_s{\beta}_2{P}_s-{\phi}_s{\beta}_3{C}_s{P}_s, $$

3 $$ \frac{\phi_l\partial {C}_l}{\partial t}=\mathbf{\nabla}\cdotp {\phi}_l{D}_C\mathbf{\nabla}{C}_l-{\phi}_l\lambda\ {C}_l-{\phi}_l{\gamma}_1{C}_l+{\phi}_s{\gamma}_2{C}_s, $$

4 $$ \frac{\phi_s\partial {C}_s}{\partial t}={\phi}_l{\gamma}_1{C}_l-{\phi}_s{\gamma}_2{C}_s, $$

where *β*_1_ [s^−1^] is the rate at which P adsorbs to soil particles, *β*_2_ [s^−1^] is the rate at which P desorbs from soil particles, *β*_3_ [m^3^ of soil solid s^−1^ μmol^−1^] is the rate associated with specific adsorption between citrate and P on soil sorption sights, *γ*_1_ [s^−1^] is the rate at which citrate adsorbs to soil particles, *γ*_2_ [s^−1^] is the rate at which citrate desorbs from soil particles, *D*_*P*_ and *D*_*C*_ [m^2^ of soil solution s^−1^] are the diffusion coefficients of P and citrate in water respectively (when *ϕ*_*s*_ > 0, these include a geometric impedance factor of 0.3), and *λ* [s^−1^] is the rate of citrate bio-degradation.

In Eqs. (1)–(4), adsorbed citrate and P are treated as separate chemical species from citrate and P in solution. The terms in Eqs. (1) and (2) with no spatial derivative describe a reversible chemical reaction between adsorbed and solution P where adsorbed citrate acts as a catalyst for P desorption. The prefactors *ϕ*_*l*_ and *ϕ*_*s*_ are included to ensure that the model accounts for changes in soil saturation, i.e. when soil saturation or soil content approaches zero the reaction terms are altered accordingly.

To solve Eqs. (1–4), a set of boundary equations is required for the microdialysis probe. The pump rate of the microdialysis probe is assumed to be fast enough so that there is an infinite supply of citrate and when P is absorbed by the microdialysis probe it is instantly transported away. It is assumed that the microdialysis probe exudes citrate at a rate proportional to the difference in concentration of citrate in the perfusate and in solution,

5 $$ {\phi}_l{D}_C\mathbf{\nabla}{\mathrm{C}}_{\mathrm{l}}\cdotp {\boldsymbol{n}}_p={\delta}_c\left({C}_0-{C}_l\right),\boldsymbol{x}\in {\Gamma}_p, $$

where *C*_0_ [μmol m^−3^ of perfusate] is the concentration of citrate in the perfusate, ***n***_*p*_(***x***) is the unit normal to Γ_*p*_ pointing out of Ω for ***x*** ∈ Γ_*p*_, and *δ*_*C*_ [ms^−1^] is the membrane permeability. In the absence of citrate, the microdialysis probe absorbs P at a rate proportional to the concentration of P in soil solution. Ionic strength of the external solution is known to alter microdialysis probe uptake rates, hence, in the model citrate can alter microdialysis probe P uptake (Demand et al. 2017). This process is not fully understood so the most general citrate dependent microdialysis probe P uptake rate is used,

6 $$ {\phi}_l{D}_P\mathbf{\nabla}{P}_l\cdotp {\boldsymbol{n}}_p=-{\delta}_P\left({C}_l\right){P}_l,\boldsymbol{x}\in {\Gamma}_{p,} $$

where *δ*_*P*_ [m of soil solution s^−1^] is a function of citrate concentration that controls microdialysis probe P uptake. In general, *δ*_*P*_ is a function of microdialysis probe membrane thickness, porosity and surface chemistry etc. To fit the data, *δ*_*P*_(*C*_*l*_) needs to be specified, but we do not know its functional form. To overcome this, *δ*_*P*_ is approximated as its first order Taylor expansion about *C*_*l*_ = 0,

7 $$ {\delta}_P={\delta}_P^0+{\delta}_P^1{C}_l+\mathcal{O}\left({C}_l^2\right) $$

Using this approximation, Eq. (6) becomes,

8 $$ {\phi}_l{D}_P\nabla {P}_l\cdot {\boldsymbol{n}}_p=-\left({\delta}_P^0+{\delta}_P^1{C}_l+\mathcal{O}\left({C}_l^2\right)\right){P}_l,\boldsymbol{x}\in {\Gamma}_{p,} $$

where ${\delta}_P^0$ has units [m of soil solution s^−1^] and ${\delta}_P^1$ has units [m^4^ of soil solution s^−1^ μmol^−1^] . The parameters ${\delta}_P^i$ are constants and can be fit using standard methods explained in the following section. The remaining parts of the boundary are closed and are described using no flux conditions,

9 $$ {D}_P\mathbf{\nabla}{P}_l\cdotp {\boldsymbol{n}}_e=0,\boldsymbol{x}\in {\Gamma}_e, $$

10 $$ {D}_C\mathbf{\nabla}{\mathrm{C}}_{\mathrm{l}}\cdotp {\boldsymbol{n}}_e=0,\boldsymbol{x}\in {\Gamma}_e, $$

where ***n***_*e*_(***x***) is the unit normal pointing out of Ω for ***x*** ∈ Γ_*e*_.Finally, the initial conditions are given by,

11 $$ {C}_l\left(0,\boldsymbol{x}\right)={C}_s\left(0,\boldsymbol{x}\right)=0, $$

12 $$ {P}_l\left(0,\boldsymbol{x}\right)=\frac{P_{add}}{\phi_l\left(1+{b}_P\right)}, $$

13 $$ {P}_s\left(0,\boldsymbol{x}\right)=\frac{\phi_l}{\phi_s}{b}_P{P}_l\left(0,\boldsymbol{x}\right) $$

where *b*_*P*_ = *β*_1_/*β*_2_ [1] is the buffer power of P in soil and *P*_*add*_ [μmol m^−3^ of total soil] is the concentration of labelled P added to the soil. Notice, after time-equilibration ${P}_s=\frac{\phi_l}{\phi_s}{b}_P{P}_l$, thus we have *ϕ*_*l*_*P*_*l*_ + *ϕ*_*s*_*P*_*s*_ = *P*_*add*_ and therefore mass is conserved in the initial conditions.

There are 9 unknown parameters introduced by the model, namely ${\beta}_1,{\beta}_2,{\beta}_3,{\gamma}_1,{\gamma}_2,\lambda, {\delta}_C,{\delta}_P^0,{\delta}_P^1$ which are summarised in Table 2. In the past, the soil reaction parameters have been estimated based purely on physical intuition (Zygalakis and Roose 2012) or have been simplified to equilibrium reaction models (i.e. they approximate sorbed P by solution P using the buffer power) by assuming the reaction rates are fast and parameterised using bulk equilibrium experiments (Gerke et al. 2000a). In order to resolve soil sorption, the reaction rate parameters must be carefully fit to experimental data. The value of *β*_3_ is of particular interest for understanding P mobilisation by citrate. However, to calculate *β*_3_ the other parameters must also be known.

**Table 2:** Parameters used in the model

Parameter	Description	Unit	Value
*ϕ*_*l*_	m^3^ of soil solution per m^3^ of total soil	m^3^ of soil solution m^−3^ of total soil	0.3 or 1
*ϕ*_*s*_	m^3^ of solid soil per m^3^ of total soil	m^3^ of soil solid m^−3^ of total soil	0.6 or 0
${b}_P=\frac{\beta_1}{\beta_2}$	Buffer power of P in soil	1	Fit
${b}_C=\frac{\gamma_1}{\gamma_2}$	Buffer power of citrate in soil	1	4.78 (Oburger et al. 2011a)
*β*_1_	P adsorption rate to solid soil	s^−1^	Fit
*β*_2_	P desorption rate from solid soil	s^−1^	Fit
*β*_3_	P enhanced desorption from soil solid due to adsorbed citrate	m^3^ of soil solid s^−1^ μmol^−1^	Fit
*γ*_1_	Citrate adsorption rate to solid soil	s^−1^	*b*_*C*_ × *γ*_2_
*γ*_2_	Citrate desorption rate to solid soil	s^−1^	Fit
*λ*	Rate of citrate biodegradation	s^−1^	Fit
*δ*_*C*_	Efflux of citrate from perfusate into the system	m^4^ of soil solution m^−3^ of total soil s^−1^	Fit
${\delta}_P^0$	Absorption rate of P by microdialysis probe when no citrate is present	m of soil solution s^−1^	Fit
${\delta}_P^1$	Absorption rate of P by microdialysis probe affected by citrate	m^4^ of soil solution s^−1^ μmol^−1^	Fit
*D*_*P*/*C*_	Diffusion rate of P or citrate in soil water	m^5^ of soil solution m^−3^ of total soil s^−1^	7 × 10^−10^ or 0.3 × 7 × 10^−10^
*t*_*max*_	Time scale of experiment	hours	1 or 12
∣Γ_*P*_∣	Surface area of the semi-permeable membrane	m^2^	5.0265 × 10^−6^
∣Ω|	Eppendorf tube volume	m^3^	1.5 × 10^−6^

### Data fitting

**Fig. 2:**
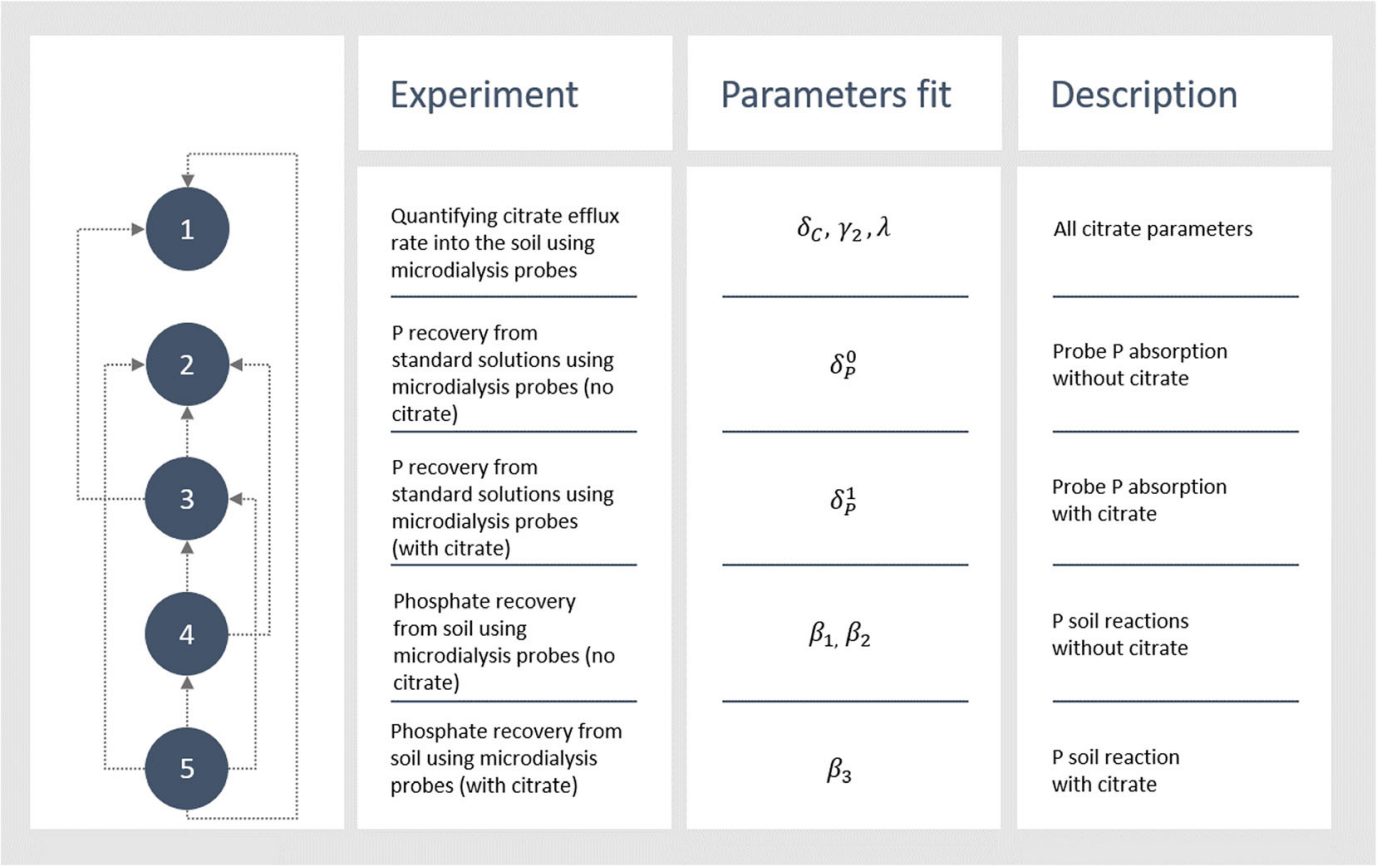
Schematic of all the data fitting procedures. The numbers dictate the order of the data fitting and the arrows represent the data fitting’s dependencies

To calculate the parameters controlling P mobilization by citrate, model parameters were varied and numerical solutions were compared to experimental results to achieve the best fit. First, the parameters controlling citrate efflux and soil reactions were fit to the results from the microdialysis organic acid calibration experiments as these parameters are independent of the P parameters. Next, the P efflux rates were fit to the results of the microdialysis P calibration results for the standard solutions as these are independent of soil reactions. Following this process, the only unknown parameters are those controlling P adsorption/desorption to soil; these were then fit to the results of the microdialysis P recovery experiments performed in soil. In each phase of the data fitting, the least-squares distance between the experimental and model values normalised by the standard deviation of the experimental data was used as an objective function. A schematic explaining the data fitting procedures is presented in Fig. 2.

Instances of the model were solved numerically on the 2D-axisymmetric domain displayed in Fig. 1, using a finite element method implemented in Comsol Multiphysics 5.3 (Comsol Ltd., Cambridge, UK). Minimisations were calculated using MATLAB 2016 (MathWorks Inc., Natick, MA) fmincon implementation of an interior-point algorithm.

### Calculating microdialysis probe citrate efflux and citrate soil reactions in this specific soil, *δ*_*C*_, *γ*_2_ and *λ*

The model was compared to the results of the experiment described in the ‘Quantifying citrate efflux rate into the soil using microdialysis probes’ experiment i.e.*,* step 1 shown on Fig. 2. As *γ*_1_ = *b*_*C*_*γ*_2_, where *b*_*C*_ is the known buffer power of citrate in soil (Oburger et al. 2011a), only one of the soil reaction parameters needed to be fit, in this case *γ*_2_. There was no P in this case, so the experiment is modelled by Eqs. (1)–(13) with P initial conditions, Eqs. (12) and (13), exchanged with,

14 $$ {P}_l\left(0,\boldsymbol{x}\right)=0, $$

15 $$ {P}_s\left(0,\boldsymbol{x}\right)=0, $$

so that there is no P in the model. If ${C}_l^{\delta_C,{\gamma}_2,\lambda; {C}_0}$is the solution to Eqs. (1)–(12) + (14), (15) with *ϕ*_*l*_ = 0.3 and *ϕ*_*s*_ = 0.6 for a given *δ*_*c*_, *γ*_2_, *λ* and *C*_0_ then the model total citrate exudation at time *t* is given by,

16 $$ {J}_{M_1}\left({C}_0,t;{\delta}_C,{\gamma}_2,\lambda \right)={\int}_0^t{\int}_{\Gamma_p}{D}_C\mathbf{\nabla}{C}_l^{\delta_C,{\gamma}_2,\lambda; {C}_0}\cdotp {\mathbf{n}}_{\mathbf{p}}d\boldsymbol{x} d\tau . $$

**Fig. 3:**
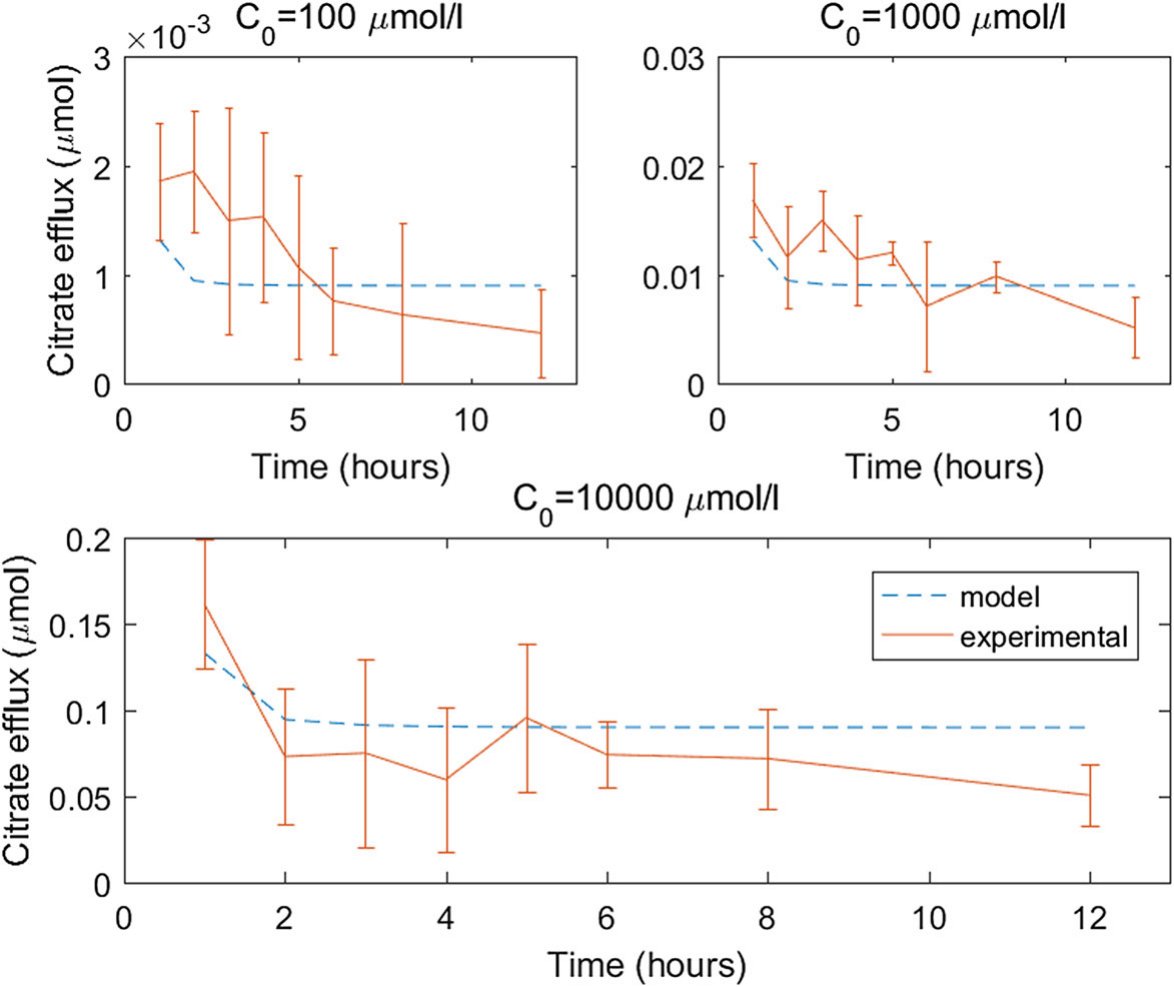
Comparison of experimental and model microdialysis probe citrate efflux using the fitted parameters δ_C_ = 4.348 × 10^−4^ ms^−1^, γ_2_ = 1.2 × 10^−2^ s^−1^ and λ = 1.1 × 10^−3^ s^−1^. The error bars on the experimental data shows standard deviation, n = 4 for C_0_ = 1000 μM, while n = 3 for C_0_ = 100 and 1000 μM

The average experimental flux at time *t* for a given concentration of citrate in the perfusate, *C*_0_, is denoted ${J}_{E_1}\left({C}_0,t\right)$ (Fig. 3), with corresponding standard deviation *σ*_1_(*C*_0_, *t*). Then *δ*_*C*_ and *γ*_2_ were found by minimising the absolute difference between ${J}_{M_1}$ and ${J}_{E_1}$. As the data is spread over a large range, it was normalised by its standard deviation. Thus, to approximate *δ*_*C*_, *γ*_2_ and *λ* the following objective function was minimised

17 $$ ob{j}_1\left({\delta}_C,{\gamma}_2,\lambda\ \right)=\sum \limits_{t\in {T}_1}\sum \limits_{C_0\in c}\frac{{\left|{J}_{M_1}\left({C}_0,t;{\delta}_{C,}{\gamma}_2,\lambda \right)-{J}_{E_1}\left({C}_0,t\right)\right|}^2}{\sigma_1{\left({C}_0,t\right)}^2}, $$

where *T*_1_ = [1, 2, 3, 4, 5, 6, 8, 12] hours and *c* = [100,1000,10000] μM.

The data fitting approach for calculating the other unknown parameters was similar to the above, as such, they are summarized in Table 3. For example, the above data fitting procedure is summarised in the first row of Table 3. As a measure of relative error of the fits, the percentage difference in cumulative exuded/absorbed citrate/P between the experiment and the model for each initial condition was reported. For example, after fitting *δ*_*C*_, *γ*_2_ and *λ* the percentage error for 100 μM of citrate in the perfusate (*C*_0_ = 100) case is

$$ \frac{J_{M_1}\left({C}_0=100,t=12 hours;{\delta}_{C,}{\gamma}_2,\lambda \right)-{J}_{E_1}\left(\mathrm{100,12} hours\right)}{J_{E_1}\left(\mathrm{100,12}\  hours\right)}\times 100. $$

**Table 3:** Data fitting details for unknown model parameters. The corresponding experimental flux to model flux ${J}_{M_i}$ is denoted ${J}_{E_i}$, *ϕ*_*l*_ = 0.3 and *ϕ*_*s*_ = 0.6 unless stated otherwise, *T*_1_ = [1, 2, 3, 4, 5, 6, 8, 12] hours, *T*_2_ = [1, 2, 3, 4, 5, 6, 7, 8, 9,10,11, 12] hours, *c* = [100,1000,10000] μM citrate, *p* = [0,100,1000,10000] μM P, ${P}_l^{p_1,\dots, {p}_N;{P}_0,{C}_0}$ is the solution to the model with parameters *p*_1_, …, *p*_*N*_ and initial conditions *P*_0_ and *C*_0_, and ${C}_l^{p_1,\dots, {p}_N;{C}_0}$ is the solution to the model with parameters *p*_1_, …, *p*_*N*_ and initial condition *C*_0_

Parameters fit	Model conditions	Model flux	Objective function
*δ*_*C*_, *γ*_2_, *λ*	*P*_*add*_ = 0	${J}_{M_1}\left({C}_0,t;{\delta}_C,{\gamma}_2,\lambda \right)={\int}_0^t{\int}_{\Gamma_p}{D}_C\nabla {C}_l^{\delta_C,{\gamma}_2,\lambda, {C}_0}\cdot {\mathbf{n}}_{\mathbf{p}}d\boldsymbol{x} d\tau$	$ob{j}_1\left({\delta}_C,{\gamma}_2,\lambda\ \right)=\sum \limits_{t\in {T}_1}\sum \limits_{C_0\in c}\frac{{\left|{J}_{M_1}\left({C}_0,t;{\delta}_{C,}{\gamma}_2,\lambda \right)-{J}_{E_1}\left({C}_0,t\right)\right|}^2}{\sigma_1{\left({C}_0,t\right)}^2}$
${\delta}_P^0$	*C*_0_ = 0, *ϕ*_*l*_ = 1, *ϕ*_*s*_ = 0	${J}_{M_2}\left({P}_0,t;{\delta}_P^0\right)={\int}_0^t{\int}_{\Gamma_p}{D}_P\nabla {P}_l^{\delta_P^0;{P}_0}\cdot {\mathbf{n}}_{\mathbf{p}}d\boldsymbol{x} d\tau$	$ob{j}_2\left({\delta}_P^0\right)=\sum \limits_{P_0\in p}\frac{{\left|{J}_{M_2}\left({P}_0,{t}^{\ast };{\delta}_P^0\right)-{J}_{E_2}\left({P}_0\right)\right|}^2}{\sigma_2{\left({P}_0\right)}^2}$
${\delta}_P^1$	*ϕ*_*l*_ = 1, *ϕ*_*s*_ = 0	${J}_{M_3}\left({P}_0,{C}_0,t;{\delta}_P^1\right)={\int}_0^t{\int}_{\Gamma_p}{D}_P\nabla {P}_l^{\delta_P^1;{P}_0{C}_0}\cdot {\mathbf{n}}_{\mathbf{p}}d\boldsymbol{x} d\tau$	$ob{j}_3\left({\delta}_P^1\right)=\sum \limits_{P_0\in p}\sum \limits_{C_0\in c}\frac{{\left|{J}_{M_3}\left({P}_0,{C}_0,{t}^{\ast };{\delta}_P^1\right)-{J}_{E_3}\left({P}_0,{C}_0\right)\right|}^2}{\sigma_3{\left({P}_0,{C}_0\right)}^2}$
*β*_1_, *β*_2_	*C*_0_ = 0 *P*_*add*_ = 66731 μmol	${J}_{M_4}\left(t;{\beta}_1,{\beta}_2\right)={\int}_0^t{\int}_{\Gamma_p}{D}_P\nabla {P}_l^{\beta_1,{\beta}_2}\cdot {\mathbf{n}}_{\mathbf{p}}d\boldsymbol{x} d\tau$	$ob{j}_4\left({\beta}_1,{\beta}_2\right)=\sum \limits_{t\in {T}_2}\frac{{\left|{J}_{M_4}\left(t;{\beta}_1,{\beta}_2\right)-{J}_{E_4}(t)\right|}^2}{\sigma_4{(t)}^2}$
*β*_3_	*P*_*add*_ = 66731 μmol	${J}_{M_5}\left({C}_0,t;{\beta}_3\right)={\int}_0^t{\int}_{\Gamma_p}{D}_P\nabla {P}_l^{\beta_3,{C}_0}\cdot {\mathbf{n}}_{\mathbf{p}}d\boldsymbol{x} d\tau .$	$ob{j}_5\left({\beta}_3\right)=\sum \limits_{t\in {T}_2}\sum \limits_{C_0\in c}\frac{{\left|{J}_{M_5}\left({C}_0,t;{\beta}_3\right)-{J}_{E_5}\left({C}_0,t\right)\right|}^2}{\sigma_5{\left({C}_0,t\right)}^2}$

When the percentage error is positive, the model over predicts exudation/absorption.

### Numerical experiments

Once the model was calibrated to the microdialysis data, simulations were carried out to answer specific scientific questions: 1) Does the microdialysis probe behave well as a model root in terms of P uptake and citrate exudation? 2) How does citrate-P solubilisation contribute to root (and microdialysis probe) P uptake? 3) Under what soil buffering and citrate biodegradation conditions is typical root citrate exudation efficient for P absorption. The following simulations were deigned to answer these questions.

### Are microdialysis probes good root analogues?

To compare microdialysis probe and root behaviour, a suitable model for a root is proposed. This was achieved by changing the boundary conditions for the microdialysis probe in the model described above to suitable equations which describe root citrate exudation and P uptake. In particular, the P boundary condition is changed from an osmosis uptake to Michaelis–Menten kinetics, as root P uptake is active and enzyme mediated (Barber 1995). The citrate boundary condition is changed to a constant rate of exudation (Geelhoed et al. 1999; Zygalakis and Roose 2012). More precisely, Eq. (5) is replaced by

18 $$ {\phi}_l{D}_C\nabla {C}_l\cdot {\boldsymbol{n}}_p={F}_C,\boldsymbol{x}\in {\Gamma}_p, $$

where *F*_*C*_ [μmol m^−2^ s^−1^] is the root citrate exudation rate; and Eq. (8) is replaced by

19 $$ {\phi}_l{D}_P\nabla {P}_l\cdot {\boldsymbol{n}}_p=\frac{-{F}_P{P}_l}{K_P+{P}_l},\boldsymbol{x}\in {\Gamma}_{p,} $$

where *F*_*P*_ [μmol m^−2^ s^−1^] is the maximum P uptake rate achieved by the root and *K*_*P*_ [μmol m^−3^] is the P concentration where the uptake rate is half *F*_*P*_. Typical exudation rates of citrate for P-starved rape roots grown in nutrient solution at 27 °C is 1.2037 × 10^−5^ μmol s^−1^ m^−1^ of root (Hoffland 1992). These roots typically have a root radius of approximately 4 × 10^−4^ m (Kjellström and Kirchmann 1994), meaning an approximate citrate exudation rate per root surface area (assuming the root is a cylinder) of *F*_*C*_ = 4.7894 × 10^−3^ μmol m^−2^ s^−1^. Typically, *F*_*P*_ = 3.26 × 10^−2^ μmol m^−2^ s^−1^ and *K*_*P*_ = 1.5 × 10^2^ μmol m^−3^ (Barber 1995). The model with Eqs. (9) and (12) will be referred to as the microdialysis probe model; when these equations are replaced by Eqs. (18) and (19) the collection of equations will be referred to as the root model. To make the models comparable, the root is assumed to have the same dimensions as the microdialysis probe.

To test whether the microdialysis probe behaves like a synthetic root in terms of citrate exudation and P uptake, the concentration of citrate in the perfusate, *C*_0_, which produces the most similar exudation rates as the model root was found using data fitting. The P uptake rates between the root model and microdialysis probe model (using the optimal *C*_0_) were then compared to determine how well the microdialysis probe behaves like a root.

### Citrate contribution to P uptake

To investigate how citrate contributes to both microdialysis probe and root P absorption, both models are solved numerically with a range of citrate exudation rates and P flux rate per surface area is plotted in time.

### Under what soil conditions and biodegradation rates is citrate important?

To determine which soil conditions citrate exudation is important for root P uptake, the root model is solved with a range of buffer powers with and without citrate exudation. The buffer power is varied by keeping desorption (*β*_2_) as the fitted value from the experiments and changing adsorption (*β*_1_). Similarly, citrate biodegradation rates were also varied. The percentage difference of P uptake between exuding and non-exuding roots over a 12 h period was plotted against buffer power.

## Results

### Experimental

The experiments in which the efflux of citrate from the microdialysis probes into soil showed that the exudation rate decays in time to reach a steady efflux rate (Fig. 3). Furthermore, when the concentration of citrate in the perfusate increased, the total quantity of citrate exuded in the soil increased linearly (data not shown).

**Fig. 4:**
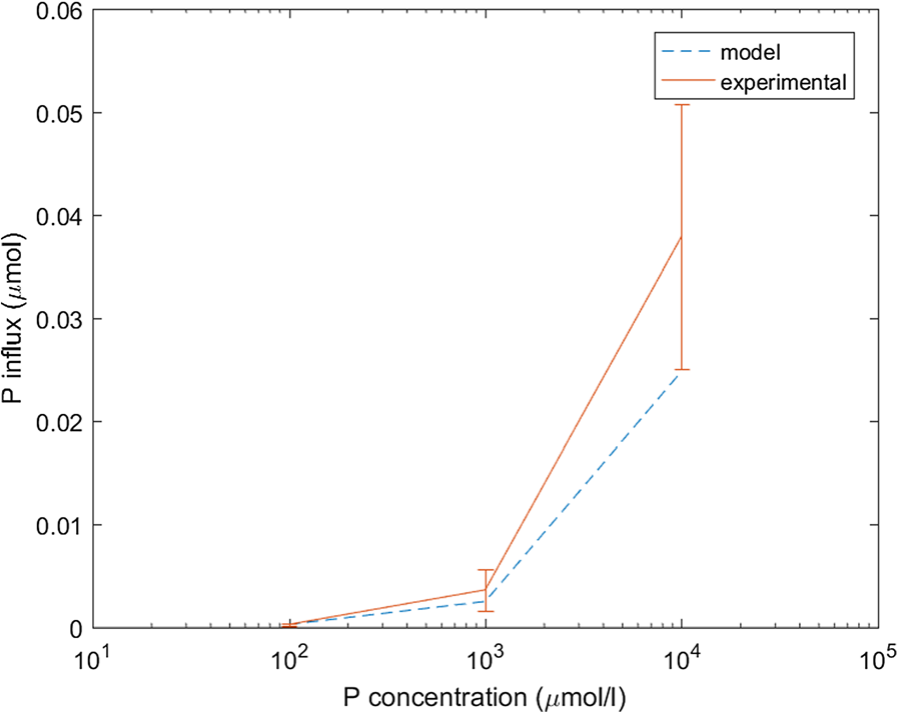
Comparison of experimental and model microdialysis probe P influx using the fitted parameter ${\delta}_{\mathrm{P}}^0=2.9357\times {10}^{-7}$ m s^−1^. The error bars on the experimental data shows standard deviation, *n* = 4, log_10_ scale on the x-axis

**Fig. 5:**
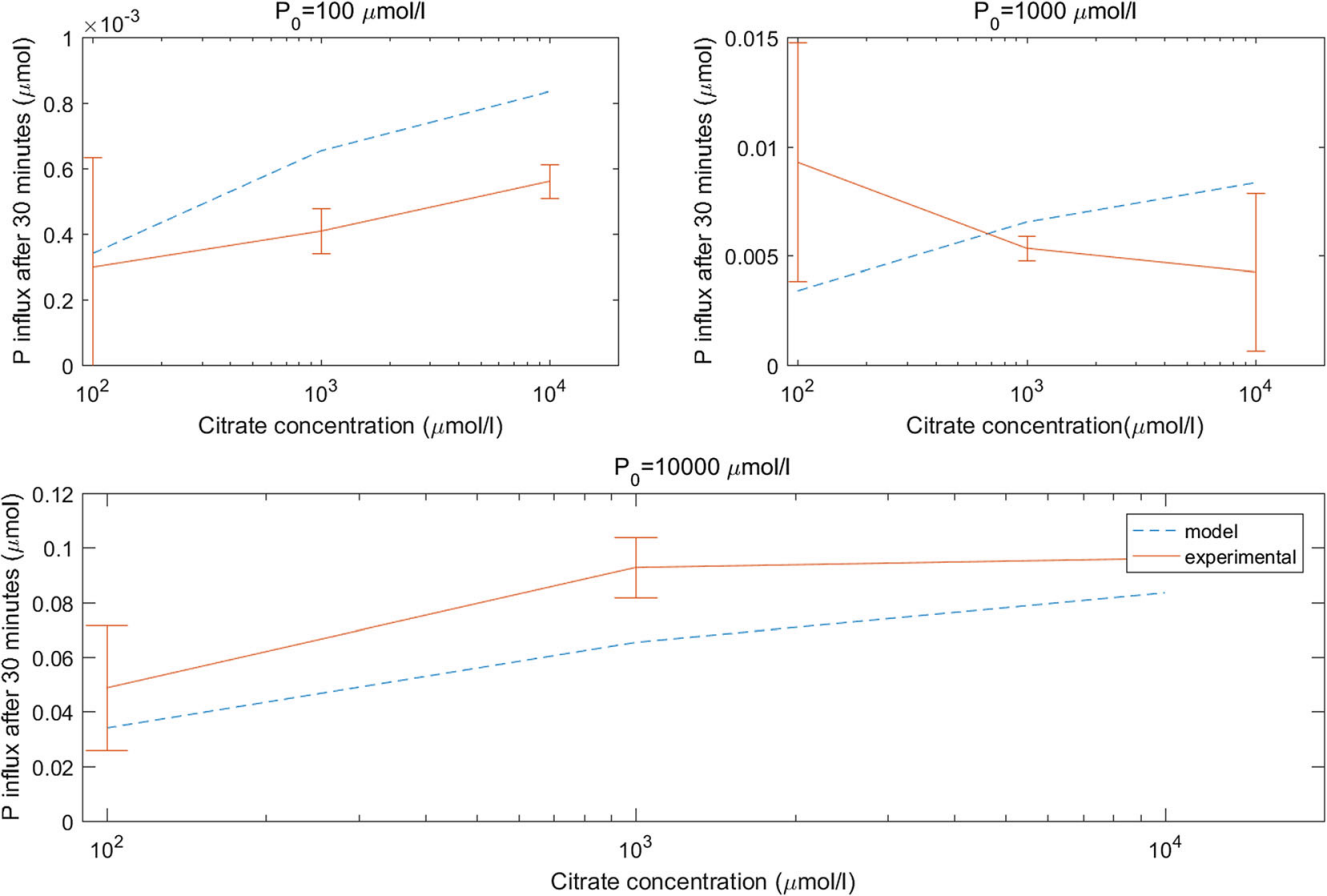
Comparison of experimental and model microdialysis probe P influx with citrate in the perfusate using the fitted parameter ${\delta}_{\mathrm{P}}^1=1.7031\times {10}^{-12}$ m^4^ s^−1^ μmol^−1^ . Error bars shows standard deviation, *n* = 4

Measurements of P recovery from standard solutions using the microdialysis probes showed that when citrate was absent in the perfusate, the quantity of P absorbed by the microdialysis probe increased linearly with the concentration of P in the standard solutions (Fig. 4). When the concentration of citrate in the perfusate increased, the amount of P absorbed by the microdialysis probe increased, except in the experiment where there was 100 μM of P in the standard solution (Fig. 5).

**Fig. 6:**
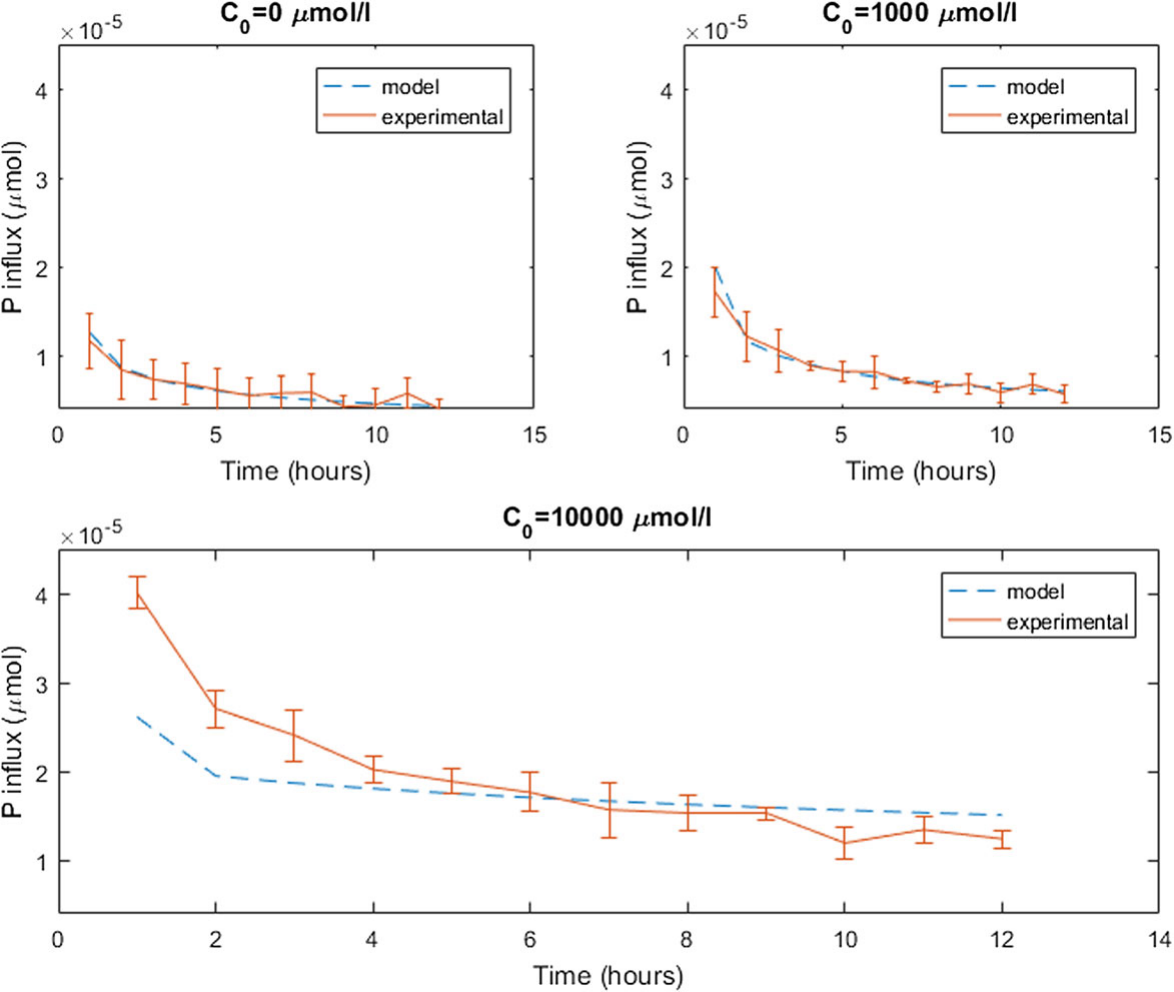
Comparison of experimental and model microdialysis probe P influx in soil with and without citrate in the perfusate. Using the parameters β_1_ = 7.899 × 10^−6^ s^−1^ and β_2_ = 1.993 × 10^−7^ s^−1^ produces the best fit to the experimental data when there is no citrate (C_0_ = 0 μM). Using the parameter β_3_ = 3.41 × 10^−13^ produces the best fit to the experimental data when there is citrate in the perfusate (C_0_ = 1000, 10000 μM). Error bars shows standard deviation, *n* = 4

When citrate was perfused through the microdialysis probes, the results showed that increasing citrate concentrations increased P recovery from the soil (Fig. 6). Furthermore, for each of the citrate concentrations in the perfusate, the quantity of P absorbed from the soil decreased over time.

### Data fitting

**Fig. 7:**
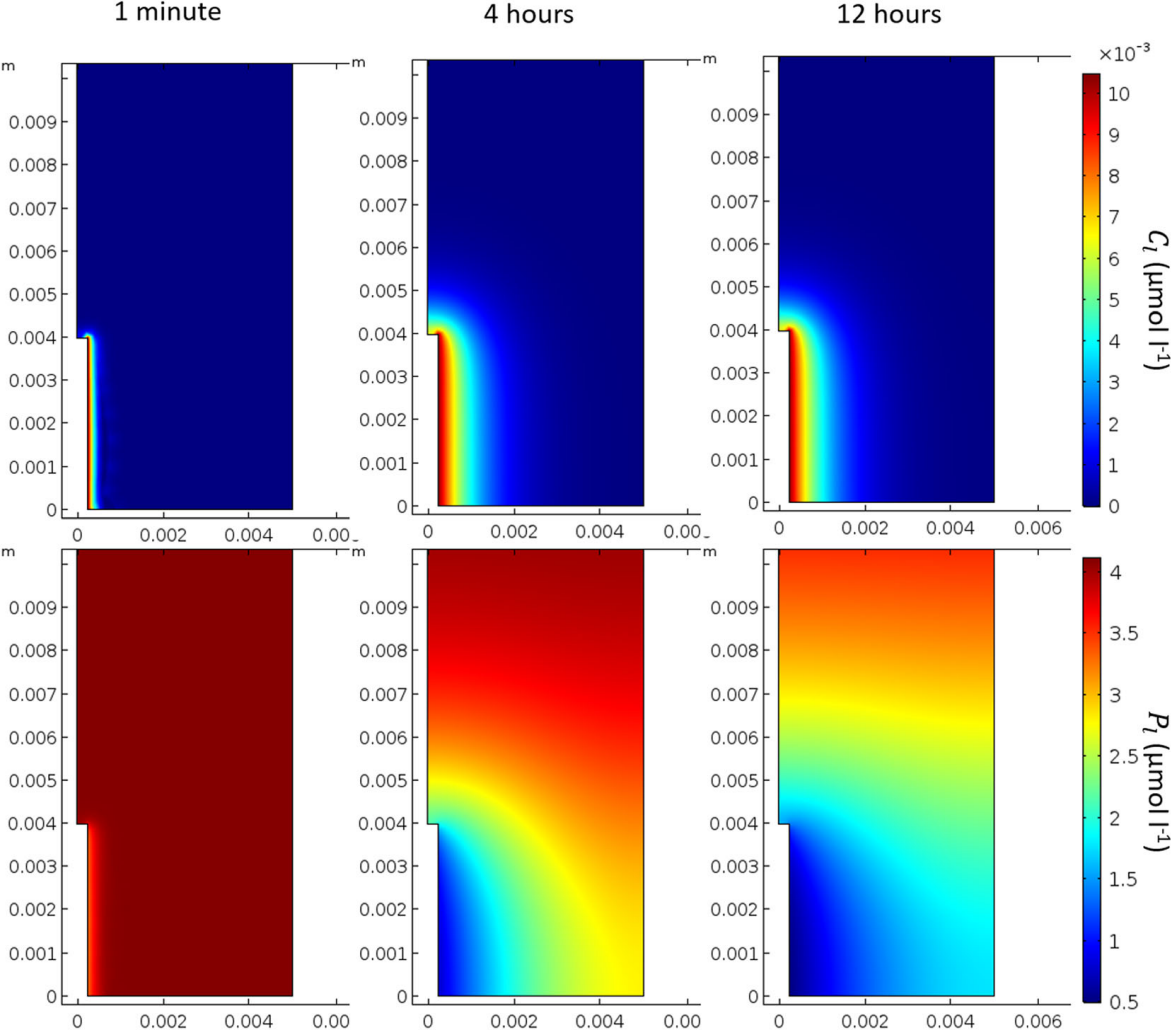
Solutions of the microdialysis probe model after 1 min, 4 h and 12 h using the fitted parameters described above with P_add_ = 6.67 μmol l^−1^ of soil and C_0_ = 10.48 μM. The top row shows the solution for citrate (C_l_) and the bottom row for phosphate (P_l_)

The value of each parameter as found by the minimisation problems described in the Data fitting section can be found in Table 4. Furthermore, the goodness of fits for minimisations 1, 2, 3, 4 and 5 as described in Fig. 2 (or Table 3) can be seen in Figs. 3, 4 and 5 (*C*_0_ = 0 μmol l^−1^), and 6 (*C*_0_ = 100, 10000 μmol l^−1^), respectively. The data fitting only concerns mass transfer across the membrane of dialysis probe. Figure 7 demonstrates the predicted distribution of P and citrate in the external soil with *P*_*add*_ = 6.67 μmol l^−1^ and *C*_0_ = 50000 μmol l^−1^ when the fitted parameters were used. The fitted parameters found in this section were used for the rest of the microdialysis probe simulations.

**Table 4:** Results of minimisations described in the data fitting section. The heading Objective function refers to functions which were minimised, details of which can be found in Table 3; argmin refers to the parameter values which achieve the minimum as found by the interior-point algorithm; Objective value shows the value of the objective function at the parameter values which achieve the minimum; and Percentage error shows percentage difference in cumulative exuded/absorbed citrate/phosphate between the experiment and the model for each initial condition, a positive value means the model over predicts the exudation/absorption

Objective function	argmin	Objective value	Percentage error
*obj*_1_(*δ*_*C*_, *γ*_2_, *λ*)	*δ*_*C*_ = 4.348 × 10^−4^ ms^−1^, *γ*_2_ = 1.2 × 10^−2^ s^−1^, *λ* = 1.1 × 10^−3^ s^−1^	30.26	*C*_0_ = 100 : − 21.4% *C*_0_ = 1000 : − 13.6% *C*_0_ = 10000 : 16.3%
$ob{j}_2\left({\delta}_P^0\right)$	${\delta}_P^0=2.936\times {10}^{-7}$ ms^−1^	349.9	*P*_0_ = 100 : − 0.02% *P*_0_ = 1000 : − 31.2% *P*_0_ = 10000:-34.5%
$ob{j}_3\left({\delta}_P^1\right)$	${\delta}_P^1=1.7031\times {10}^{-12}$ m^4^ s^−1^ μmol^−1^	110.7	*P*_0_ = 100, *C*_0_ = 100:-14% *P*_0_ = 100, *C*_0_ = 1000:60% *P*_0_ = 100, *C*_0_ = 10000:49% *P*_0_ = 1000, *C*_0_ = 100:-63% *P*_0_ = 1000, *C*_0_ = 1000:22% *P*_0_ = 1000, *C*_0_ = 10000:95% *P*_0_ = 10000, *C*_0_ = 100:-30% *P*_0_ = 10000, *C*_0_ = 1000:-29% *P*_0_ = 10000, *C*_0_ = 10000:-13%
*obj*_4_(*β*_1_, *β*_2_)	*β*_1_ = 7.899 × 10^−6^ s^−1^, *β*_2_ = 1.993 × 10^−7^ s^−1^	1.2	*C*_0_ = 0:-1.7%
*obj*_5_(*β*_3_)	3.41 × 10^−13^ m^3^ of soil solid s^−1^ μmol^−1^	100.8	*C*_0_ = 1000 : 1.6% *C*_0_ = 10000 : − 8.7%

### Numerical experiments

#### Microdialysis probes as model roots

**Fig. 8:**
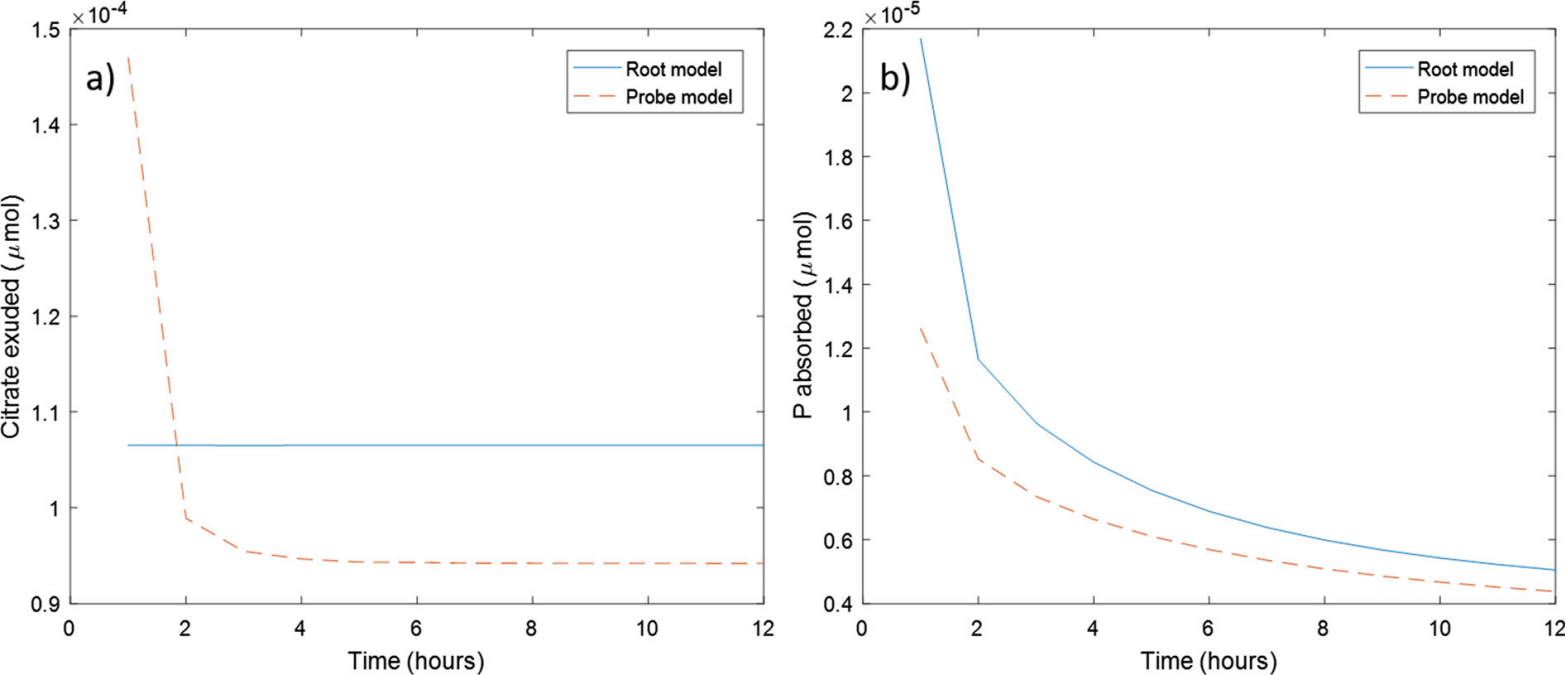
Comparison of the model root and model microdialysis probe in terms of citrate exuded and P absorbed using the concentration of citrate in the perfusate which produces the most similar citrate exudation to a typical root (C_0_ = 10.48 μM). a) Root and microdialysis probe model citrate exudation measured every hour; b) Root and microdialysis probe model P absorption measured every hour

It was found that a citrate concentration of 10.48 μM in the perfusate produced the most similar citrate exudation pattern to a model rape root (Fig. 8a). The P absorption for microdialysis probe and root model was also compared using the same initial P additions as the *β*_1_, *β*_2_ and *β*_3_ data fitting (*P*_*add*_ = 66731 μmol m^−3^ of total soil) Fig. 8b. It was found that at this initial concentration of P in the soil and citrate in the perfusate, the microdialysis probe under predicts root P absorption.

#### Citrate’s contribution to P uptake

**Fig. 9:**
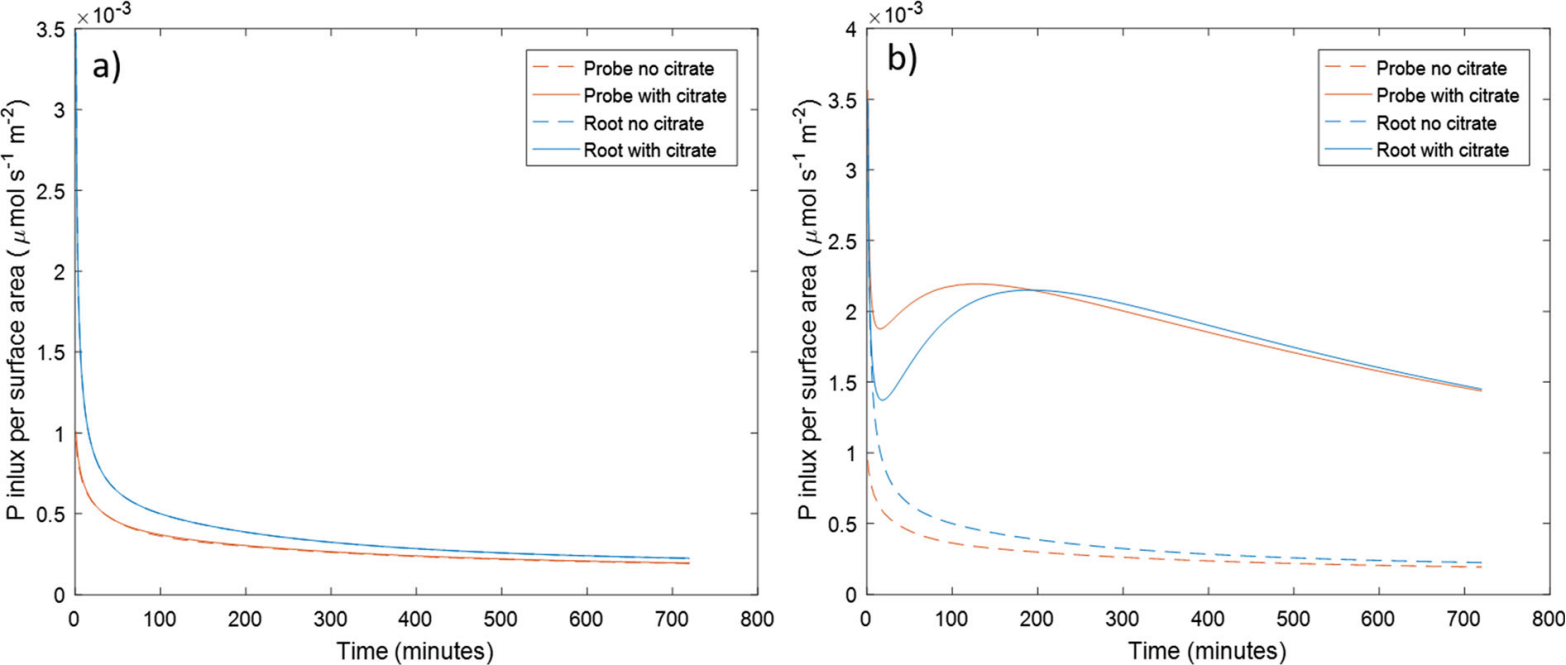
P influx per surface area in a model root and microdialysis probe with and without citrate exudation. a) In the microdialysis probe model the concentration of citrate in the perfusate is C_0_ = 10.48 μM, which produces similar citrate exudation to the root model with exudation rate F_C_ = 4.7894 × 10^−3^ μmol m^−2^ s^−1^, typical for a rape root. The no exudation cases overlap the exudation cases. b) In the microdialysis probe model the concentration of citrate in the perfusate is C_0_ = 50000 μ*M*, which produces similar exudation to the root model with exudation rate F_C_ = 21.25 μmol m^−2^ s^−1^

**Fig. 10:**
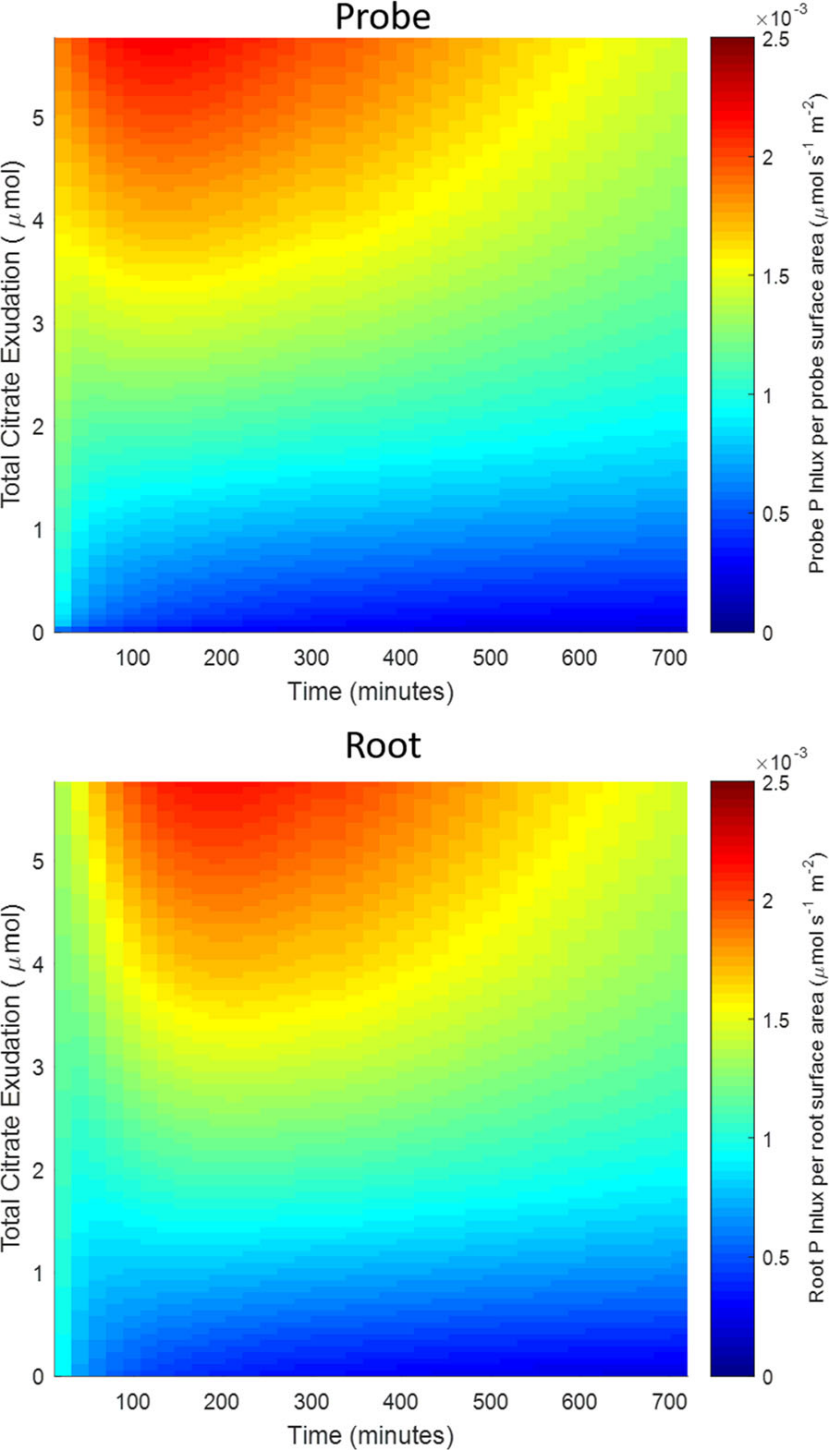
Heat map showing P influx per surface area against time and total amount of citrate exuded into the soil over 12 h for both a model root and microdialysis probe. The increasing exudation total are evaluated by solving the Probe and Root models with increasing values of *C*_0_ and *F*_*c*_ respectively

The P uptake rate per surface area of both a model microdialysis probe and root exuding citrate (*C*_0_ = 10.48 μ*M*, *F*_*C*_ = 4.7894 × 10^−3^ μmol m^−2^ s^−1^) was compared to those with no citrate (Fig. 9a). Similarly, Fig. 9b shows the effect on P uptake when citrate exudation is dramatically increased (with *C*_0_ = 50000 μM, and *F*_*C*_ = 21.25 μmol m^−2^ s^−1^ to produce similar root and microdialysis probe citrate exudation). Little difference in P absorption between a model exuding microdialysis probe/root (exuding at a typical rate for plants) and non-exuding microdialysis probe/root could be seen (<1%). Figure 10 shows P influx versus time for a range of citrate exudation rates. Microdialysis probe and root P uptake dynamics remained similar for a range of citrate exudation quantities. Furthermore, uptake dynamics changed drastically as more citrate was exuded into the soil. When enough citrate is exuded into the soil, large increases in P influx can be obtained for both a root and microdialysis probe.

#### Under what soil conditions is citrate important?

**Fig. 11:**
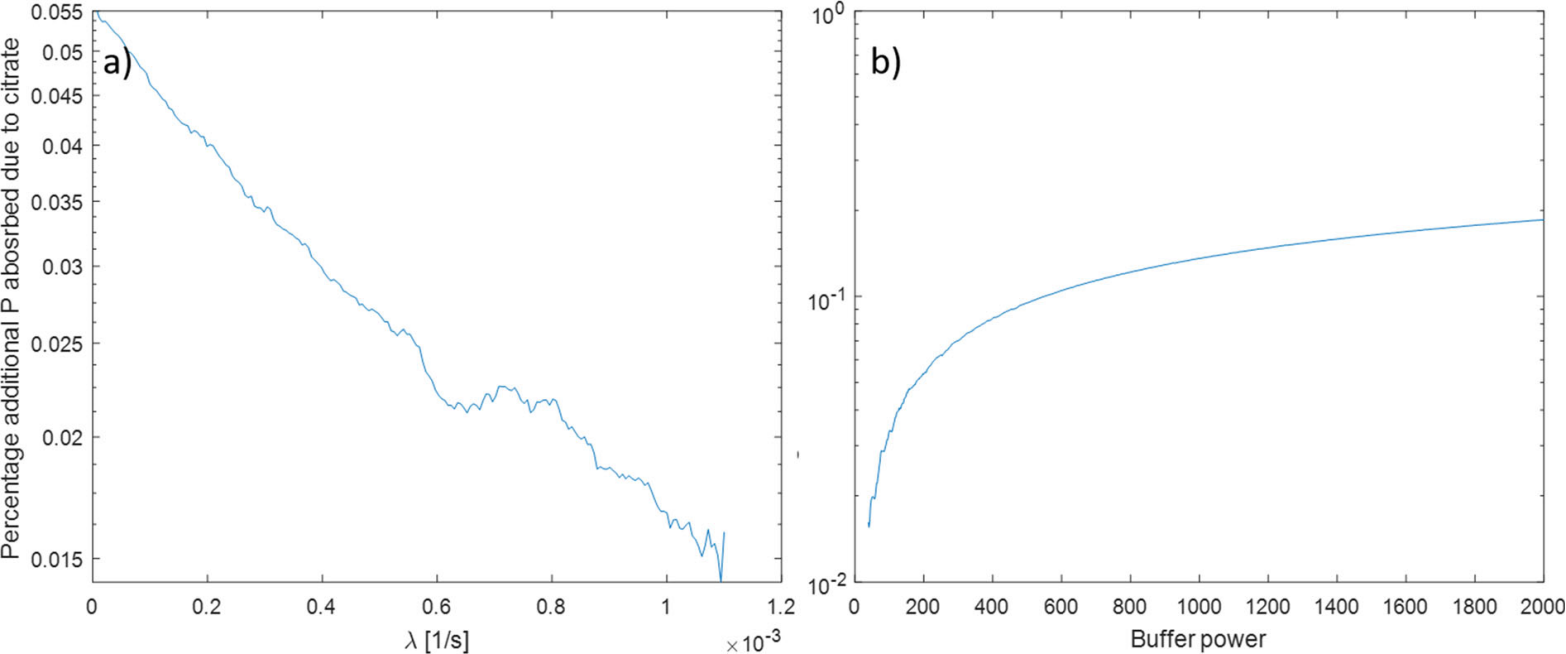
Plots of percentage additional P absorbed due to citrate when compared to a non-exuding root, a) when citrate biodegradation, λ was increased from 0, b) when P buffer power, b was increased from 39

The buffer power and biodegradation rate were varied to determine which soil conditions citrate exudation is important for P absorption. Figure 11a shows the percentage difference in P absorbed when comparing an exuding root to a non-exuding root when the citrate biodegradation rate is varied. Figure 11b shows the same when buffer power is varied. Percentage additional P absorbed decreases exponentially in citrate biodegradation and increases linearly in buffer power (notice the y axis in Fig11 are logarithmic). Error from the numerical scheme is evident due to the small relative changes (relative error of the method is at most 0.01%).

## Discussion

### Modelling P mobilisation by citrate in soil

The model proposed here introduces a parameter (*β*_3_) which controls the rate of mobilisation of soil phosphate by citrate. This was similar to parameters found in many other soil P mobilisation models (Gerke et al. 2000a; Zygalakis and Roose 2012). Of critical importance, however, was that we were able to experimentally derive this key parameter which had only previously been estimated from intuition. Our model fitted well to the dynamic data from the ‘P recovery from soil using microdialysis probes’ experiment when the concentration of the citrate in the perfusate was 0 or 1000 μM, and it fitted well to the cumulative behaviour of the probe when the concentration was 10,000 μM. Thus, we conclude that the mechanism of citrate enhanced P-desorption assumed in the model is consistent with experiments and could account for the enhanced P influx by the microdialysis probe for a limited range of citrate and P concentrations. In particular, as the model uses first order kinetics to model sorption, it is not suitable for long-term modelling were P concentrations vary (Dari et al. 2015). To improve the suitability of the model for a wide range concentrations, the first order kinetics should be expanded upon, when more data emerges, to include the non-linear relationship between citrate and P concentration with sorption and citrate enhanced P desorption. Oburger et al. (2009) calculated the bio-degradation rate of citrate in a similar soil to be *λ* = 6.87 × 10^−5^ [s^−1^] by measuring CO_2_ respiration, while our calculation was *λ* = 1.1 × 10^−3^ [s^−1^]. However, Oburger et al. (2009) used a double first order exponential decay model to fit the data, while we used a single first order decay model and considered soil adsorption. We also ascribe this difference to the significant temporal decoupling which can occur between substrate uptake and mineralization which leads to an underestimation of λ using the CO_2_-based approach (Gunina et al. 2017). However, our approach measured biodegradation and sorption together while the CO_2_ respiration approach targets microbial activity. Furthermore, Fig. 11a suggests such changes in biodegradation only makes small changes to the amount of P absorbed and hence would not be detectable by microdialysis.

### Data fitting

In total, 8 parameters were fit to 72 data points with varying citrate and P conditions and time resolution. The data fitting approach was to determine the dependency of the parameters, as seen in Fig. 2, then design experiments suitable for determining the parameters which had the least dependencies. The parameters dependent on multiple processes could then be fit to experiments. This approach allowed us to decouple parameters effects from one another. For example, if all parameters were fit together, P desorption (*β*_2_) could increase at the expense of citrate enhanced solubilisation (*β*_3_) and citrate enhanced desorption would be underestimated (or vice versa).

Although the cumulative exudation of citrate over the 12 h period in the model is within 21% of the experimental values for each citrate concentration (Table 4), there were mechanisms regarding probe citrate exudation that the model was not capturing (see Fig. 3). Notably, the experiments showed that probe citrate exudation slows gradually, while the microdialysis probe model rapidly decreased to reach a steady state efflux rate. Time-dependent probe permeability could explain this. There are also many citrate processes in the soil that are not included in the model, such as microbial mineralisation and immobilization of citrate and microbial population dynamics that could account for the poor temporal fit to the data (Glanville et al. 2016). These processes were not included in the current model as they were not measured in the experiments. Additional micro-dialysis probe experiments, such as citrate flux in water and citrate recovery from soil are required to determine which mechanism to include and fit the parameters reliably. However, as the cumulative error is relatively small, the effect on the subsequent data fitting procedures will be minimal.

Poor fits were achieved when fitting citrate enhanced probe P uptake (${\delta}_P^1$, Table 4, Fig. 5), this was assigned to two possible causes: 1) Inconsistent and variable experimental data; 2) Linearizing *δ*_*P*_(*C*) about *C*_*l*_ = 0 (eq. 7) incurred a larger error. Linearizing about *C*_*l*_ = *C*_0_ may be more suitable in the future when only one concentration of citrate in the perfusate is used. However, in our model, including this mechanism was important for the subsequent fitting of citrate enhanced solubilisation (*β*_3_). If citrate enhanced probe P uptake was not included then the additional absorbed P due to citrate altering probe osmosis rates would be attributed to citrate solubilising P, and *β*_3_ would have been overestimated. Although the correction has errors, the following results are more precise rather than having not included the correction.

### Microdialysis probes as root analogues

After the soil P parameters were derived, a root model was proposed to determine if the microdialysis probes can be used to mimic root behaviour under the soil conditions detailed in Table 1, and the P additions stated in the experimental section. The microdialysis probe was found to underestimate root P uptake, with the difference narrowing as time progressed. We attribute this to the P supply rapidly depleting adjacent to the root, putting the Michaelis–Menten kinetics into the linear range of P concentrations. The probe under predicts root uptake as the linearized root uptake rate constant is $\frac{F_P}{K_P}=2.17\times {10}^{-4}$ m s^−1^ while probe permeability is ${\delta}_P^0=2.9\times {10}^{-7}$ m s^−1^. Using a linearized Michaelis–Menten expression is only valid for a small range of P concentrations near 0. It was shown experimentally that microdialysis probe exudation rate decayed over time while we assumed the root exuded citrate at a constant rate, as evidenced by other authors (Geelhoed et al. 1999; Schnepf et al. 2012; Zygalakis and Roose 2012). Constant root exudation could be realistic as a large electrochemical potential gradient exists between the root and soil which can drive citrate exudation even against a large external concentration (Jones 1998). This contrasts with the microdialysis probe where citrate exudation is solely driven by the strength of the diffusion gradient and associated ion sieving effects at the microdialysis probe-soil interface (Galach and Waniewski 2012). Our findings, however, suggest that a suitable concentration of citrate can be used in the perfusate so that the microdialysis probe exudes the same quantity of citrate as a model root in total, but fails to mimic the dynamic behaviour.

### Modelling the impact of organic acid exudation on root P uptake

When we used a citrate exudation rate similar to an oilseed rape plant (Hoffland 1992) and added 0.1 μmol of P to the model soil, it was found that little additional P was absorbed compared to a non-citrate exuding root (<1% enhancement of P acquisition). In comparison, other models report significant gains from citrate exudation. After 16 days of model time, Schnepf et al. (2012) found an entire root system could gain between 4 and 19% extra P depending on exudation patterns. Schnepf et al. (2012) used the kinetic competitive Langmuir reaction equation (Van de Weerd et al. 1999) they assumed desorption was fast to send citrate-enhanced P solubilisation in equilibrium (the parameters were not experimentally verified) and considered multiple roots which interacted. They also used a root exudation rate of 3 × 10^−2^ μmol m^−2^ s^−1^, an order of magnitude larger than that in the current study. Zygalakis and Roose (2012) used a similar model of citrate-enhanced P solubilisation to that used in the current work, with the reactions sent into equilibrium. They used a *β*_3_/ *β*_1_ ratio two orders of magnitude larger than the current (with no experimental support) and predicted cluster roots can absorb up to 35% more P due to citrate exudation. However, assuming the soil reactions are fast relative to diffusion to send the soil reactions into equilibrium can incur an error. Using a similar non-dimensionalisation to Zygalakis and Roose (2012) (non-dimensionalise P concentration with *K*, sorbed citrate concentration with the maximum achieved when using the realistic root exudation rate (${C}_s^{max}$) and length, *l*, with the height of the Eppendorf tube) we find that adsorption happens at rates on the order of 10^0^ ($\frac{\beta_1{l}^2}{D}$) desorption at 10^−1^ ($\frac{\beta_2{l}^2}{D}$) citrate enhanced desorption 10^−5^ ($\frac{\beta_3{l}^2{C}_S^{max}}{D}$) and diffusion 10^0^ ($\frac{D}{D}$) when in regions of high citrate concentrations and using the fitted parameters. Thus, assuming reactions are fast relative to diffusion in the current geometry would not be valid. However, as the size of the geometry, *l*, increases assuming the reactions are in equilibrium becomes more appropriate. In contrast, previous experiments are in agreement with the current finding, both Güsewell and Schroth (2017) and Ryan et al. (2014) could not detect P uptake gains in high carboxylate exuding plants in comparison to low carboxylate exuding subgenus/near-isogenic species.

When citrate exudation was increased incrementally, P uptake dramatically increased (Figs. 9 and 10) and P uptake reached a distinct maximum at approximately 250 min when citrate exudation reached appropriate levels (Fig. 10). The latter effect is attributed to a solubilisation peak caused by P mobilisation. This mimics experiments performed in this same soil where high soil citrate concentrations (10 mM) were needed to promote plant ^33^P uptake (Khademi et al. 2010; Palomo et al. 2006).

The reader should be aware that the model may not be as accurate for very high concentrations of citrate as suggested by the relatively poor fit to the temporal experimental data in Fig. 6, *C*_0_ = 10000 μM. In contrast, the fit for *C*_0_ = 1000 μM case was good. This is a manifestation of the error from the linear approximations of P/citrate soil reactions (the error is $\mathcal{O}\left({C}_l^2\right)$ or $\mathcal{O}\left({P}_l^2\right)$ i.e. the error increases as concentration increases). Citrate-enhanced solubilisation of P speeds up with increased citrate concentration and a Langmuir-like isotherm for citrate/P adsorption and citrate enhanced solubilsation may be needed to capture the temporal behaviour for both citrate concentrations simultaneously. The error from the linearized kinetics may also become apparent when the P and citrate concentrations vary due to the probe absorbing and exuding. However, we cannot justifiably include the non-linear terms as the current experiments do not measure these concentration dependent effects. In future work additional experiments with varying P concentration in the soil could be used to fit the additional parameters which control these mechanisms. This would require a series of further microdialysis experiments designed to investigate citrate/P adsorption and desorption for varying concentrations.

Although citrate concentrations in the bulk soil are typically <50 μM, concentrations up to 10 mM have been reported in the rhizosphere for certain plants under P deficiency (Dessureault-Rompré et al. 2006), the current parameterisation of the model may not be accurate for such high concentrations. In addition, the model only considers 4 mm of a single root exuding, while cluster roots, or roots in close proximity may act together to exude larger quantities of citrate. From Fig. 10, we estimate that a plant would need to exude citrate at a rate of 0.73 μmol cm^−1^ of root h^−1^ to see a significant increase in P absorption. Alfalfa (*Medicago sativa* L.) can exude 1.3 μmol of citrate g^−1^ of dry root d^−1^ when under P stress (Lipton et al. 1987), which equates to approximately 1.4 × 10^−5^ cm^−1^ of root h^−1^ (Solaiman et al. 2007), orders of magnitude lower than the required rate. Hence, P gains could only be achieved if the roots were densely packed. This concurs with the modelling findings of Zygalakis and Roose (2012) and Gerke et al. (2000b) who both found that large clusters of roots benefit most from citrate exudation. The rates calculated in this work could be used to parametrise image-based models to assess different root system architectures, such as cluster roots, and the utilization of solubilized P. Gerke et al. (2000a) found that more than 10 μmol of citrate g^−1^ soil was needed for a significant increase in P solubilisation using bulk-equilibrium experiments. Gerke et al. (2000a) did not see P solubilisation with lower citrate concentrations, however, this does not necessarily imply that plants would fail to see enhanced P uptake as seen in this work (Fig. 10) for a number of reasons. Firstly, the calculation of *β*_3_ in this work suggests that the rate citrate solubilises phosphate is in fact slow and should be considered dynamically; fast-equilibration arguments to approximate adsorbed P by P in solution would not stand, nor would equilibrium experiments be representative of a root absorbing P. Furthermore, citrate is exuded from a root creating a local region of high concentration, allowing the dramatic citrate-phosphate solubilisation as seen in Gerke et al. (2000a) near the root surface.

Unsurprisingly, when citrate biodegradation decreases, the percentage additional P absorbed by the root due to citrate increases, however, the importance of this was less than some other factors in the model. For example, when the value of the buffer power was increased and P becomes held more strongly on the solid phase, citrate exudation had more benefit at solubilising P. This agrees with both the experimental work of Zhang et al. (1997), who suggest that low-molecular weight organic acids help radish (*Raphanus sativus* L.) and rape (*Brassica napus* L.) utilize sparingly soluble P; and the modelling work of Schnepf et al. (2012) who found citrate solubilised more P in strongly sorbing soils.

The *β*_1_ and *β*_2_ parameters calculated in this study results in a buffer power of 39.6 for phosphate in this soil. Although this was not unreasonable for such a soil and P additions (Barber 1995), some caution is required when interpreting this result. Firstly, the microdialysis probe was only calibrated for P influx in standard solutions, however, when the microdialysis probe was placed in soil, the ionic strength of the soil may have altered the uptake rate of the microdialysis probe. During the data fitting, this effect was included in the *β*_1_ and *β*_2_ parameters and may not be representative of the actual buffer power. This could be overcome by calibrating the probes at a similar ionic strength and compositions as exists in the soil. Furthermore, large quantities of P added to the soil can lower the buffer power (Barber 1995) and the scintillation counting used in this paper only measured the isotopically labelled P added to the soil, not the P originally present in the soil. However, these artefacts were accounted for during the *β*_1_ and *β*_2_ data fitting as ionic strength affects were measured implicitly during the corresponding experiment, thus will not affect the *β*_3_ data fitting. Similarly, any gains in P uptake by the probe due to acidification by the un-buffered citrate was attributed to *β*_3_, the parameter controlling specific adsorption, during the data fitting.

## Conclusions

Here we demonstrated that microdialysis can be used to provide an effective measure of the diffusive flux of solutes both into and out of soil. The microdialysis probes can be easily used to mimic root exudation. Their small size and rapid response time makes them ideal to detect the spatial and temporal dynamics of solutes at the soil-root interface. We also demonstrated that assumptions about mechanisms of citrate and P in bulk soil can be used to create a model which describes the recovery of P by the probes. Parameters in this model were then varied so that the model fluxes across the microdialysis probe membrane were consistent with microdialysis experiments, allowing accurate measurements (up to the validity of the assumptions made) of soil properties. Critically, we show the importance of calibrating the microdialysis probe influx and efflux rates in separate specially designed experiments to correct for the sensitivity of the microdialysis probes to external factors. This approach proved effective in calculating citrate-enhanced P desorption and may be useful in calculating other important dynamic plant-soil interactions.

## Electronic supplementary material

ESM 1 (DOCX 97 kb)

ESM 2 (JPG 82 kb)
