# Supplementary material for: Quantifying citrate-enhanced phosphate root uptake using microdialysis
Source: Plant Soil. 2019 Dec 5;461(1-2):69–89. doi: 10.1007/s11104-019-04376-4 (PMC8550755; doi:10.1007/s11104-019-04376-4)
Supplement: Supplementary file 1 — (DOCX 97 kb) [file 11104_2019_4376_MOESM1_ESM.docx]

Supplementary Material: Quantifying citrate-enhanced phosphate root uptake using microdialysis

# Basics of Microdialysis Probes

Microdialysis probes consist of a semi-permeable membrane with plumbing to allow water to travel around the probe from an inlet to an outlet (Fig. S1). The ‘perfusate’ is the solution which is fed into the probe and the ‘dialysate’ is what is collected. As the perfusate passes through the microdialysis probe, solutes from the external solution can pass across the semi-permeable membrane into the probe, which is then collected. Similarly, solutes in the perfusate can pass from the probe into the external solution. The relative recovery (the ratio of the concentration of solute in the dialysate to the external solution) is increased as the flow rate decreases as the exchange time along the membrane increases.

| 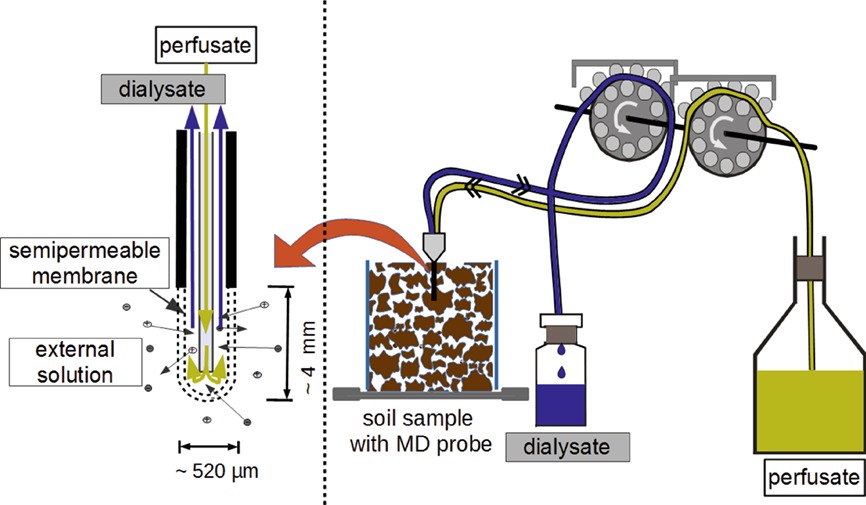 |
| --- |
| **Fig. S1** Diagram explaining microdialysis nomenclature and operation. Figure taken from (Demand et al. 2017). |

# References

Demand D, Schack-Kirchner H, Lang F (2017) Assessment of diffusive phosphate supply in soils by microdialysis. Journal of Plant Nutrition and Soil Science 180, 220-230.
