# Supplementary figures and images for: Quantifying citrate-enhanced phosphate root uptake using microdialysis
Source: Plant Soil. 2019 Dec 5;461(1-2):69–89. doi: 10.1007/s11104-019-04376-4 (PMC8550755; doi:10.1007/s11104-019-04376-4)

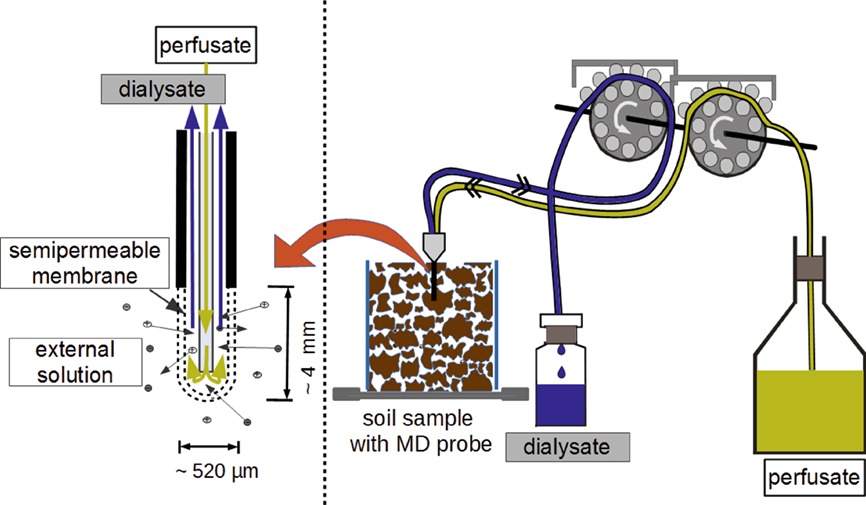

Supplement: Supplementary file 2 — (JPG 82 kb) [file 11104_2019_4376_MOESM2_ESM.jpg]
